# Unveiling Hidden Mercury and Methylmercury Sources: The Role of Submarine Groundwater Discharge in Coastal Lagoons

**DOI:** 10.1021/acs.est.5c07191

**Published:** 2025-09-15

**Authors:** Céline Lavergne, Júlia Rodriguez-Puig, Clara Ruiz-González, María Montero-Curiel, Gemma Casas, Daniel Romano-Gude, Irene Alorda-Montiel, Júlia Dordal-Soriano, Aaron Alorda-Kleinglass, Marc Diego-Feliu, Javier Gilabert, Alex Campillo-de La Maza, Cristina Romera-Castillo, Natalia Torres-Rodriguez, Lars-Eric Heimbürger-Boavida, Jordi García-Orellana, Valentí Rodellas, Andrea G. Bravo

**Affiliations:** † Departament de Biologia Marina i Oceanografia, 58341 Institut de Ciències del Mar (ICMCSIC), 08003 Barcelona, Spain; ‡ HUB Ambiental UPLA, Departamento Ciencias y Geografía, Facultad de Ciencias Naturales y Exactas Universidad de Playa Ancha, 2340000 Valparaiso, Chile; § Institut de Ciència i Tecnologia Ambientals, 16719 Universitat Autònoma de Barcelona, 08193 Bellaterra, Spain; ∥ Departament de Física, Universitat Autònoma de Barcelona, 08193 Bellaterra, Spain; ⊥ Department of Ocean & Earth Sciences, Old Dominion University, Norfolk, Virginia 23529, United States; # Department of Civil and Environmental Engineering (DECA), 16767 Universitat Politècnica de Catalunya, 08034 Barcelona, Spain; 7 Department of Chemical and Environmental Engineering, Universidad Politécnica de Cartagena, 30202 Cartagena, Spain; 8 Mediterranean Institute of Oceanography (MIO), Aix Marseille Université, CNRS/INSU, Université de Toulon, IRD, 13009 Marseille, France

**Keywords:** biogeochemistry, mercury budget, methylmercury, submarine groundwater discharge, coastal aquifer, Mar Menor

## Abstract

Mercury loads from Submarine Groundwater Discharge (SGD) may represent an overlooked source of methylmercury (MeHg) to the ocean, affecting human and ecosystem health. The SGD process involves the flow of fresh, saline, or mixed groundwater from coastal aquifers into the oceans classified in different components. Existing studies rarely report the fluxes supplied by the different SGD components; therefore, the relevance of SGD as a source of mercury remains unclear. We aimed to quantify SGD-driven mercury/methylmercury fluxes to the coast, focusing on the largest coastal lagoon in the western Mediterranean. We measured total dissolved mercury and MeHg in surficial and porewaters during the summer and autumn. Porewaters were enriched in total dissolved Hg and MeHg compared to the lagoon. Lagoon shore waters and porewaters with a high concentration of labile dissolved organic matter were prone to MeHg formation and thus had higher MeHg concentrations. The mercury input through SGD to the Mar Menor lagoon (4300 mmol year^–1^) was similar to atmospheric deposition and 1 order of magnitude greater than the stream input. Among different components of the SGD, long-scale lagoon water recirculation dominated. These findings have substantial implications for the regional Hg budget and raise awareness of the importance of considering the different SGD components for an accurate estimation of SGD-based Hg input to the coastal ocean.

## Graphical abstract

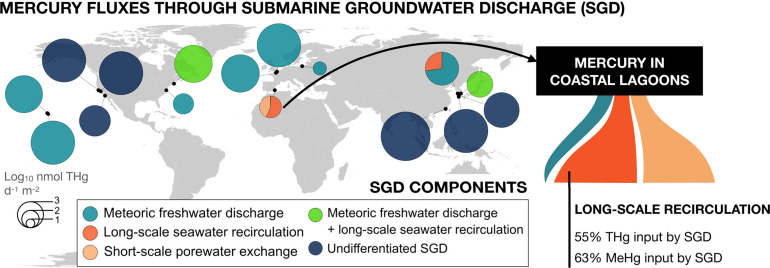

## Introduction

Coastal groundwaters are sensitive land-ocean transition zones,^1,2^ hydraulically connected to the sea, facilitating the exchange of water between the sea and the aquifer—either through seawater intrusion into the aquifer or Submarine Groundwater Discharge (SGD) from the aquifer to the sea. While SGD is commonly classified into five categories, it can be grouped into three components based on the origin of the discharging groundwater, the driving forces behind the flow, and the geological characteristics of coastal aquifers namely: meteoric groundwater discharge, long-scale seawater recirculation, or short-scale porewater exchange (Figure 1). The fluxes from SGD are highly variable at a global scale, mostly depending on the type of the aquifer and the coast (e.g., rocky, sandy, or muddy shorelines), the anthropogenic pressure in the watershed, and marine forcing.^3–5^ SGD is widely recognized as a significant source of inorganic and organic nutrients, inorganic carbon, dissolved organic matter, metals, among others both at local and regional scales.^6–8^

**Figure 1:**
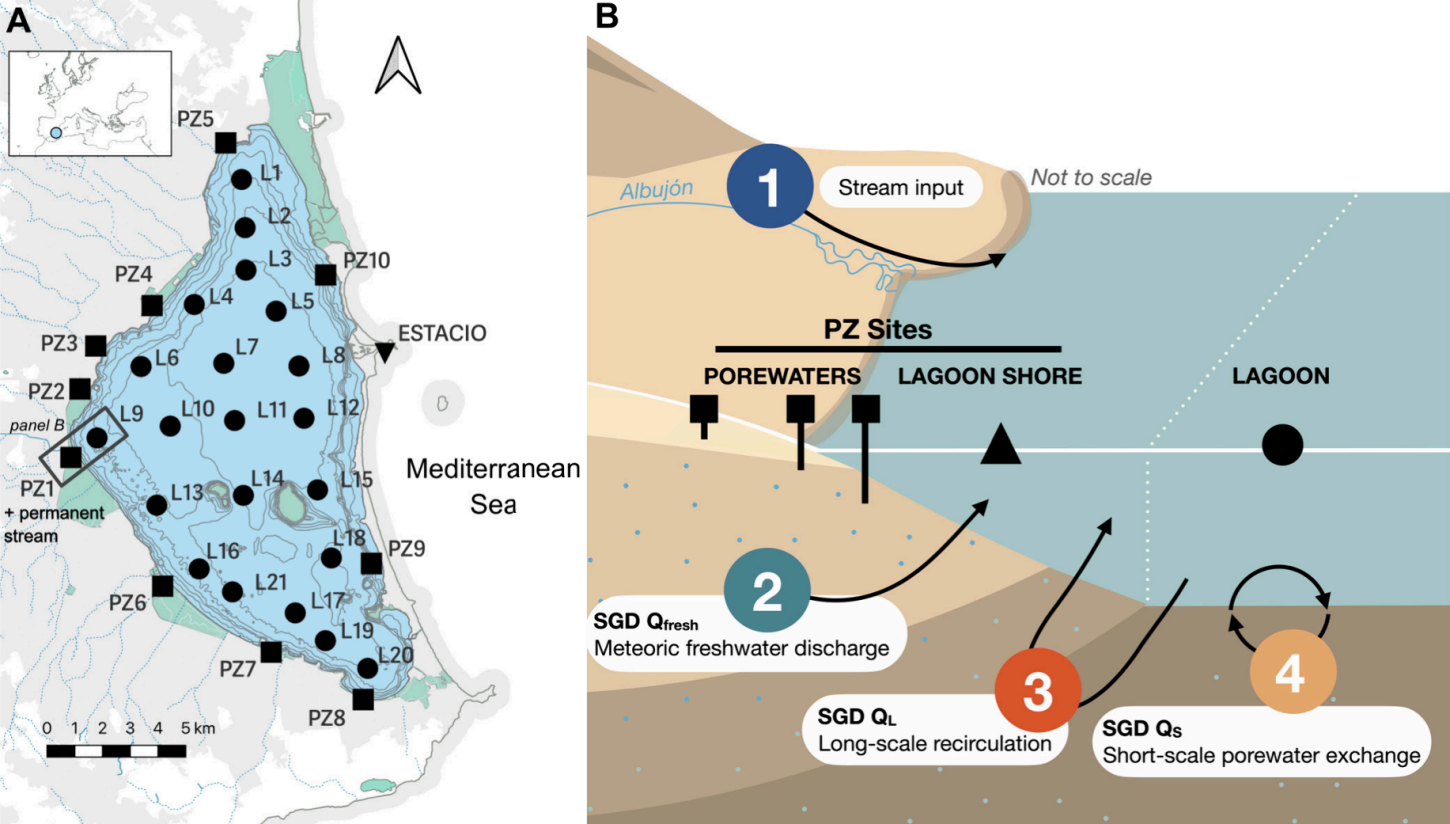
Mar Menor coastal lagoon. A) Location of the sampling sites. The green area represents the wetland areas provided by the Spanish Ministry for Environment. B) Schematic representation showing the different types of samples collected and the 4 sources of Hg studied in the current study including the main SGD components. Lagoon shore samples represented with a turned-up triangle in panel B cannot be visualized in panel A for its proximity to the piezometer sites.

Mercury (Hg) is a nonessential, persistent metalloid, the organic methylmercury (MeHg) being the most neurotoxic form with the ability to bioconcentrate, bioaccumulate, and biomagnify in the trophic web.^9–11^ The formation of MeHg seems to be controlled by Hg­(II) bioavailability^12,13^ but also determined by organic matter (OM, composition and concentration),^14–17^ by physicochemical conditions such as pH, temperature, oxidation state of sulfur and iron species, as well as by the activity and composition of a diverse group of anaerobic bacteria and archaea harboring the *hgcA/B* gene cluster, used as a biomarker for Hg methylating prokaryotes.^18–21^ Due to the mixing of fresh groundwater from the aquifer with infiltrated seawater, these land–ocean transition zones are often sites of intense biogeochemical activity, characterized by pronounced physicochemical gradients.^5,22^ Due to the influence of anthropogenic sources affecting aquifers, coastal groundwaters often have high nutrient concentrations, low oxygen and high dissolved OM (DOM) concentration of multiple origins and lability.^4,7,23^ Coastal groundwaters also harbor diverse and active microbial communities adapted to hypoxic conditions,^24^ making them potential hotspots for MeHg production, which can therefore reach the coastal zone via SGD. However, only 10 reports are available to date concerning the role of SGD in Hg cycling,^25–34^ and none have focused on its impact in coastal lagoons.

Previous studies demonstrated that total Hg and/or MeHg discharge to coastal areas through SGD may be equivalent or exceed the atmospheric deposition in Massachusetts,^34^ California^28,33^ (USA), Jeju Island^29^ (Korea), Hampyeong Bay^30^ and Jiulong River estuary^31^ (China). In contrast, other authors reported lower SGD-driven Hg inputs relative to atmospheric deposition or rivers in the southern Baltic Sea.^32^ The discrepancies regarding the role of SGD as a relevant pathway for Hg to the coastal ocean, combined with the limited studies investigating the contributions of specific SGD components underscore the urgent need for a more thorough understanding of SGD’s function as a Hg transporter at the land–ocean interface. This is particularly crucial given that global^35^ and regional^36^ Hg budgets often overlook the importance of this process.

To fill this knowledge gap, our study aimed at decrypting the role of SGD as a potential source of total Hg (total dissolved Hg) and MeHg for coastal areas, using the coastal lagoon Mar Menor (Spain) as an example. Total dissolved Hg and MeHg have not yet been assessed in Mar Menor water or coastal groundwater. By measuring total dissolved Hg and MeHg during autumn and summer in different SGD components, our study provides new insights into the role of SGD, showing an enrichment of Hg and MeHg in SGD relative to lagoon waters and deciphering the biogeochemical drivers enhancing MeHg concentrations in coastal areas. Notably, among the different SGD components, long-scale lagoon water recirculation was identified as the primary source of Hg to the coastal lagoon throughout the year.

## Materials and Methods

### Study Site

The Mar Menor is a shallow hypersaline coastal lagoon, with an average depth of less than 6 m, separated from the Mediterranean Sea by a sandbar called “La Manga”. It is a semienclosed water body of 135 km^2^, only connected to the sea by three small inlets. It acts as a receptor of terrestrial inputs of dissolved compounds and hence represents a suitable site to study Hg/MeHg contribution from coastal groundwater. This eutrophic coastal lagoon is mainly impacted by acid-mine drainage, cattle industry/agriculture, and urban/touristic activities.^37,38^ The Mar Menor recently experienced anoxic events potentially linked to brine discharges from desalination plants, wastewater discharges, and oxygen-depleted SGD. Situated in a semiarid region with Mediterranean climate, the evaporation highly exceeds precipitation of 300 mm year^–1^ on average.^39^ While agriculture irrigation is intense, the lagoon presents high seasonal variability of temperature^40,41^ and receives limited surface water inputs with a single permanent stream flowing into the lagoon (the Albujón stream). Annual average surface runoff to the Mar Menor is estimated to be on the order of 50 hm^3^ year^–1^, greatly variable depending on the amount of precipitation and the intensity of precipitation events.^42^ Most of the inputs from surface runoff occur through the Albujón permanent stream, with discharge flows generally ranging from 1 × 10^4^ to 1.5 × 10^5^ m^3^ day^–1^ (values retrieved from the Canal Mar Menor streamflow monitoring database for the period of study; https://canalmarmenor.carm.es/). Estimates of sediment inputs through the Albujón stream are on the order of 1 × 10^4^ t year^–1^.^43^

The average of water renewal time of the lagoon ranges between 150 and 400 days^44,45^ and varies according to the location, the year and the season.^46^ An unconfined Quaternary shallow coastal aquifer is connected to the lagoon, through which fresh groundwater discharges at rates from 11 to 68 × 10^6^ m^3^ year^–1^.^42,47–49^

### Sampling Strategy

Main sampling was performed in 10 sites (Figure 1) during summer and autumn, two contrasting periods in terms of precipitations^39^ and SGD water fluxes:^50^ 13–20th July 2021 and 16–23rd November 2021. Filtered porewaters were collected near the shoreline using PTFE-based piezometers coupled to a Masterflex L/S PTFE-Diaphragm Pump System connected to an acid-washed 47 mm filter PFA Tefzel Clamp in which a sequential filtration was performed using preburnt GF/D (2.7 μm), GF-A (1.6 μm), and GF/F (0.7 μm) glass fiber filters (Whatmann, USA). When possible, porewaters were collected at different depths ranging from 10 to 80 cm (*n* = 32; “Porewaters; Figure 1). Surface water of the lagoon shore was directly collected near each piezometer site (*n* = 17; “Lagoon shore”; Figure 1), and surface water from the lagoon was collected at 0.5 m depth following a Kriging scheme with sampling sites separated by <2 km (*n* = 41; “Lagoon”; Figure 1). All surface waters were collected and filtered using PTFE tubing connected to the Masterflex L/S PTFE head pump and to the sequential PFA filtration clamp. Additional samples were collected in the permanent stream (*n* = 1) and in the Mediterranean Sea at the main inlet of the lagoon, called Estació (*n* = 2; Figure 1).

### Total Dissolved Hg and Methylmercury (MeHg) Concentrations in Water Samples

Analytical methods for total Hg and MeHg are detailed in Supporting Methods 1. Briefly, the total dissolved Hg and dissolved MeHg (MeHg) (*n* = 92 and *n* = 67, respectively) were measured in all water samples previously acidified on site to 0.4% v/v (HCl Ultrex II, J. T. Baker, USA). Total dissolved Hg was measured following a modified version of the USEPA1631 method (US EPA, Method 1631, 1999) based on cold vapor atomic fluorescence spectrometry (CV-AFS, Brooks Rand Model III, USA) coupled to a custom-made semiautomatic single gold trap.^51^ The limit of detection was 0.03 pM. MeHg was measured by double-spiking species specific isotope dilution gas chromatography (GC, THERMO GC 1300 with GC220 transfer module) coupled to a sector field ICP-MS (Thermo Element XR) (DSIDA-GC-SF-ICP-MS).^52^ Briefly, the samples were spiked with solutions enriched in Hg isotopes as follow: 0.08–2.24 pg g^–1^ inorganic ^199^Hg and 0.01–4.52 pg g^–1^ Me ^201^Hg to ensure a robust quantification with optimal excess ratios.^53^ Detection limit was 0.002 pM.

### Ancillary Parameters

The analytical methods used to measure ancillary parameters are detailed in Supporting Information. It includes physicochemical parameters, dissolved organic carbon (DOC), nutrients, fluorescent dissolved organic matter (FDOM) characterization, radium isotopes, and bulk prokaryotic heterotrophic activity.

### Determination of Total Dissolved Hg and MeHg Fluxes

#### Water Flow

The SGD and stream flows to the lagoon (m^3^ day^–1^) were estimated with the combination of a radium mass balance for ^224^Ra_,_^226^Ra, and ^228^Ra and a regional hydrogeological model of the lagoon for the two seasons.^50^ This multimethod approach allowed distinguishing between the three main components of SGD occurring in the system (Figure 1): *Q*_F_ which is the flow associated with the meteoric discharge of fresh groundwater, *Q*_L_ referring to the long-scale recirculation of lagoon water, and the *Q*_S_ corresponding to the short-scale porewater exchange.^54^ As detailed in Rodriguez-Puig et al.,^50^ long-scale recirculation involves processes occurring over longer temporal scales, whereas short-scale recirculation includes processes such as porewater exchange operating over time scales of minutes to days.

#### Calculation of the Total Dissolved Hg and MeHg SGD-Driven Fluxes

The total dissolved Hg and MeHg fluxes are simply obtained by multiplying the water flow of each SGD component by the total dissolved Hg or MeHg concentration in the discharging groundwater (endmember). Daily fluxes were expressed in mmol or μmol day^–1^ (for total dissolved Hg and MeHg, respectively) and annual fluxes were expressed as mol year^–1^. For comparison purposes, details are available in the Supporting Information (Table S5) including the values normalized by the total area of the studied lagoon (i.e., 135 km^2^).

#### Endmember Definition

The conversion of SGD water flows into SGD-driven Hg fluxes requires known concentrations of Hg species in the groundwater discharged by each SGD component (i.e., the SGD endmembers; Cook et al.^55^). To assess the uncertainty of the fluxes, an endmember corresponded to the median, and the first and third quartiles (median and interquartile range) of total dissolved Hg and MeHg measured concentrations for every SGD component and was defined within the set of samples available (Table S2).

#### Endmember for QF

The endmember corresponded to the total dissolved Hg (or MeHg) concentration measured in coastal porewaters with salinity <10 psu.

#### Endmember for *Q*_L_ and *Q*_S_

These fluxes are recirculation of lagoon waters; hence, the endmembers for a net flux calculation corresponded to the total dissolved Hg (or MeHg) concentration measured in discharging porewaters minus the mean concentration of total dissolved Hg (or MeHg) from the lagoon. In particular, for *Q*_L_, the discharging porewater corresponded to saline porewaters with salinity >29.5 psu. For *Q*_S_, this recirculation of lagoon water is likely a ubiquitous process occurring at the lagoon water-sediment interface. Hence, the discharging porewaters were defined as the porewater of surficial lagoon sediments (0–6 sediment depth). Although we did not measure total dissolved Hg and MeHg concentrations in the porewater of lagoon surface sediments, porewater concentrations at lagoon surface sediments were calculated using sediment concentrations and partitioning coefficients reported in the literature (Supporting Methods 1 and Table S3). Briefly the equations used were

$$\begin{array}{l} C_{\left[THg\;Dissolved\right]}=C_{\left[THg\;Particulate\right]}*\log_{10}K_{D}\\ C_{\left[MeHg\right]}=C_{\left[THg\right]}*\%MeHg \end{array}$$

with *C*_[THg Dissolved]_ being the concentrations of total dissolved Hg in the surficial porewaters of the lagoon calculated using *C*_[THg Particulate]_ the mean concentration of total particulate Hg measured in the first 6 cm of lagoon sediments (140 ± 82 ng total Hg g^–1^ dry sediment; Alorda-Montiel et al.^56^). The median (interquartile range) partitioning coefficient (log_10_ *K*_D_) was 5.27 ± 0.9 L kg^–1^ based on a literature review (*n* = 36; Table S3). The uncertainty due to the partitioning coefficient was assessed and included in the calculation by applying the formula with the median log_10_ *K*_D_, the first quartile log_10_ *K*_D_ and the third quartile log_10_ *K*_D_. The % MeHg was the mean percentage of MeHg relative to total dissolved Hg found in the saline coastal porewaters (4.0 ± 5.2).

### Statistical and Multivariate Analyses

Statistical analyses and figures were performed using R CRAN version 4.3.1 ^57^ with R studio environment version 2023.06.2. Data are summarized as median ± the standard error. Significant influence of the sampling period and type of water on the different parameters was determined through a type III two-way ANOVA for unbalanced design followed by the Tukey–Kramer posthoc test. Based on non-normality distribution of the data, nonparametric Spearman correlation tests were performed to reveal linear relationships between variables. To decipher the biogeochemical patterns among the different studied sites and periods, a principal component analysis (PCA) was conducted from 92 observations and a selection of 10 features based on *a priori* importance and collinearity using the *PCA* function of the ‘FactoMineR’ package.^58^ Significant clustering in the ordination by sampling period was tested by permutational two-way ANOVA (PERMANOVA) after checking the homogeneity within groups (*betadisper* function; vegan package^59^). The clustering by water type did not pass the homogeneity assumption; hence, PERMANOVA was not applied. To identify the main drivers of MeHg concentrations and % MeHg, backward and forward selections were applied to first select the best explicative variables based on the AIC values. The best explicative variables selected by both models were used to build the final explanatory linear model. To estimate the relevance of SGD inputs into the lagoon, the SGD-derived Hg inventory was calculated and contrasted to the inventory of Hg in the lagoon according to Rodellas et al.^60^ (detailed in Supporting Methods S3).

## Results and Discussion

### Contrasting Geochemical Signatures along the Terrestrial-Marine Continuum and Across Seasons

A multivariate analysis was performed with the ancillary parameters to facilitate the visualization of the main biogeochemical signatures of the studied samples and showed clustering by sampling period and type of water (Figure 2). The first two components of the PCA accounted for 55.2% of the total variation within the data set.

**Figure 2:**
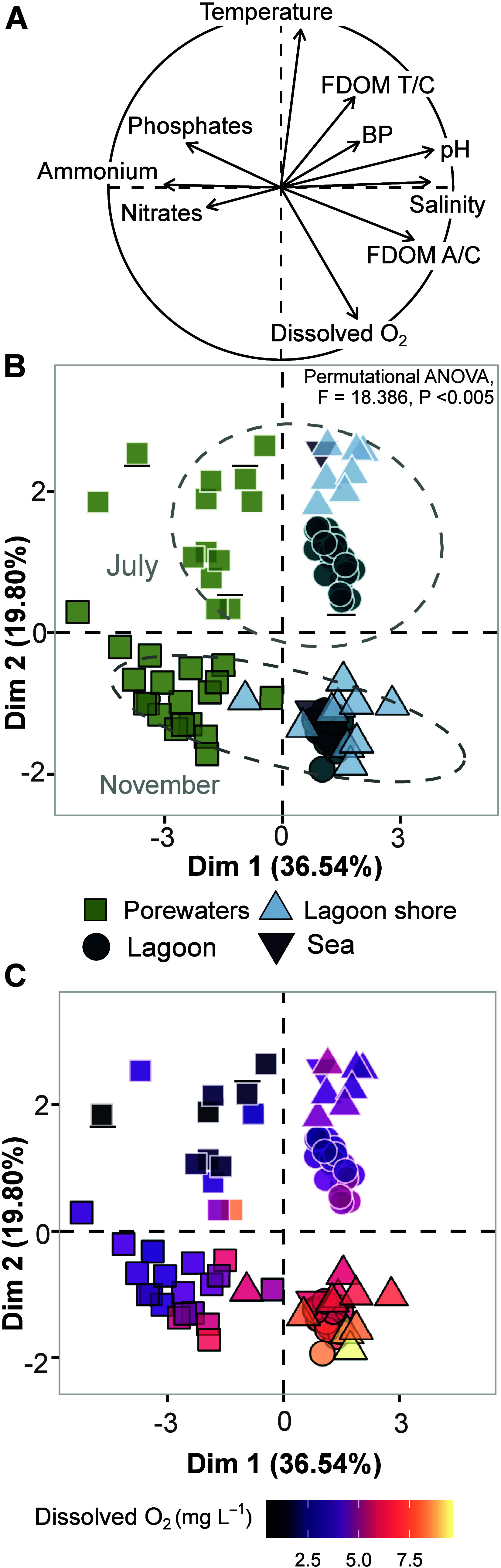
Principal component analysis (PCA) of geochemical data in both July and November from porewaters, lagoon shore, lagoon waters, and sea. A) Correlation circle showing the contribution of each variable (*n* = 10) in the ordination. “BP” stands for prokaryotic heterotrophic activity, “FDOM T/C” is the ratio of peak T and peak C for fluorescent dissolved organic matter and “FDOM A/C” is the ratio of peak A and peak C values. B) Individual map of 92 observations. Samples are colored by water type, and the presence of white or black border represented the sampling period (July and November, respectively). C) Same individual map of 92 observations using the same shapes and borders, colored by the dissolved oxygen concentration (mg L^–1^).

The first component (accounting for 36.9% of variation; Figure 2) discriminated the different water types from terrestrial to marine waters. Porewaters were distinguished by higher concentrations of nutrients such as ammonium (NH_4_^+^), nitrate (NO_3_^–^), and phosphate (PO_4_^2–^), as well as terrestrial fresh humic-like fluorescent dissolved organic matter (FDOM) characterized by lower A/C ratios. Elevated nitrate concentrations (>300 μM, which exceed previous observations range^47^) were mainly observed at urban and agricultural sites (porewaters from PZ5, PZ3, and PZ7), likely stemming from anthropogenic contamination infiltrating through runoff.^61^ Contrastingly, the highest ammonium concentrations were recorded in porewaters from wetland areas (PZ2 and PZ4), where the elevated levels of dissolved organic carbon (DOC) reaching up to 3390 μM suggest a natural origin linked to the rich organic matter in these regions.^61^ Lagoon waters, in contrast, showed increased levels of degraded humic-like FDOM, indicated by higher aromatic substances (peak A) to humic-like substances (peak C) A/C ratios (*P* < 0.05), reflecting a distinct biogeochemical signature compared to porewaters.

The second component (explaining 18.3% of the variability) separated the samples from the two distinct periods of the year (July and November; PERMANOVA, *F* = 18.386, *P* < 0.01). Regarding the ratio of fluorescence peaks T/C, the highest values were observed in July in lagoon shore waters, indicating an enrichment of protein-like substances, likely linked to a higher microbial activity (Figure 2A and Figure S2). While all studied water samples were more oxygenated in November, compared to July, porewaters exhibited a greater degree of oxygen depletion in comparison to lagoon and lagoon shore waters (ranging from 1.2 to 6.5 mg L^–1^; Figure 2C and Figure S1), with 28% of the samples classified as hypoxic (defined as concentrations below 2.5 mg L^–1^ ^62,63^ ). Low oxygen concentrations and the median oxidation–reduction potential (ORP) measured in porewaters (−89.4 ± 21.4 mV, Figure S2) are characteristic of iron and, specially, sulfate reduction conditions.^61,64^

Hence, this PCA ordination enables one to track the terrestrial influence of SGD along the first dimension and the monitoring of seasonal variability along the second dimension; revealing the presence of pronounced spatial and seasonal variations in the biogeochemical signatures of the examined waters.^40,65^

### SGD Reaching the Largest Western Europe Lagoon Is a Source of Total Dissolved Hg

The concentration of total dissolved Hg ranged from 0.43 to 120 pM, displaying a decline along the terrestrial–marine continuum. The porewaters exhibited elevated total dissolved Hg concentrations (median: 5.13 ± 4.37 pM; Table S4) relative to both the lagoon shore and lagoon waters (median: 2.91 ± 1.04 and 1.17 ± 0.09 pM, respectively; *P* < 0.05; Figure 3A). While total dissolved Hg concentrations have only been assessed in sediments, eels and macrophytes in the Mar Menor,^56,65–67^ our values from porewaters are comparable to the reported range of <16 to 61.3 pM in coastal groundwaters from the Waquoit Bay (USA)^34^ and higher than the concentrations found in the Bay of Puck (<5.7 pM; Poland).^32^ Total dissolved Hg concentrations were significantly correlated to both ^224^Ra and the first PCA component. Ra-224 inputs to the Mar Menor lagoon are mainly supplied by SGD, so both ^224^Ra and the first PCA component are proxies indicating the influence of terrestrial inputs, and particularly SGD, to lagoon waters (Figure S4, Rodellas et al.^68^). This correlation suggests that SGD acts as a source of dissolved total dissolved Hg to the restricted Mar Menor lagoon as also shown in the sandy permeable coastal aquifer of the Waquoit Bay (USA)^34^ and in the open areas of the California coast.^33^ This finding contrasts with that observed in the Bay of Puck, where seawater exhibited the highest total dissolved Hg concentrations compared to porewaters. In the Mar Menor lagoon, SGD was a source of total dissolved Hg throughout the year. Indeed, considering all types of studied waters there was no significant difference in total dissolved Hg concentrations between July and November (type III two-way ANOVA; *F* = 2.448, *P* = 0.12), and the correlations of total dissolved Hg with SGD proxies (^224^Ra and PCA1) were valid in both July and November (*P* < 0.05; Figure S5). Finally, the concentrations of total dissolved Hg measured in the Mediterranean Sea at the exit of the lagoon ranged from 1.45 to 1.61 pM (Figure 3B) and are only slightly higher than those reported for the Mediterranean Sea.^36^

**Figure 3:**
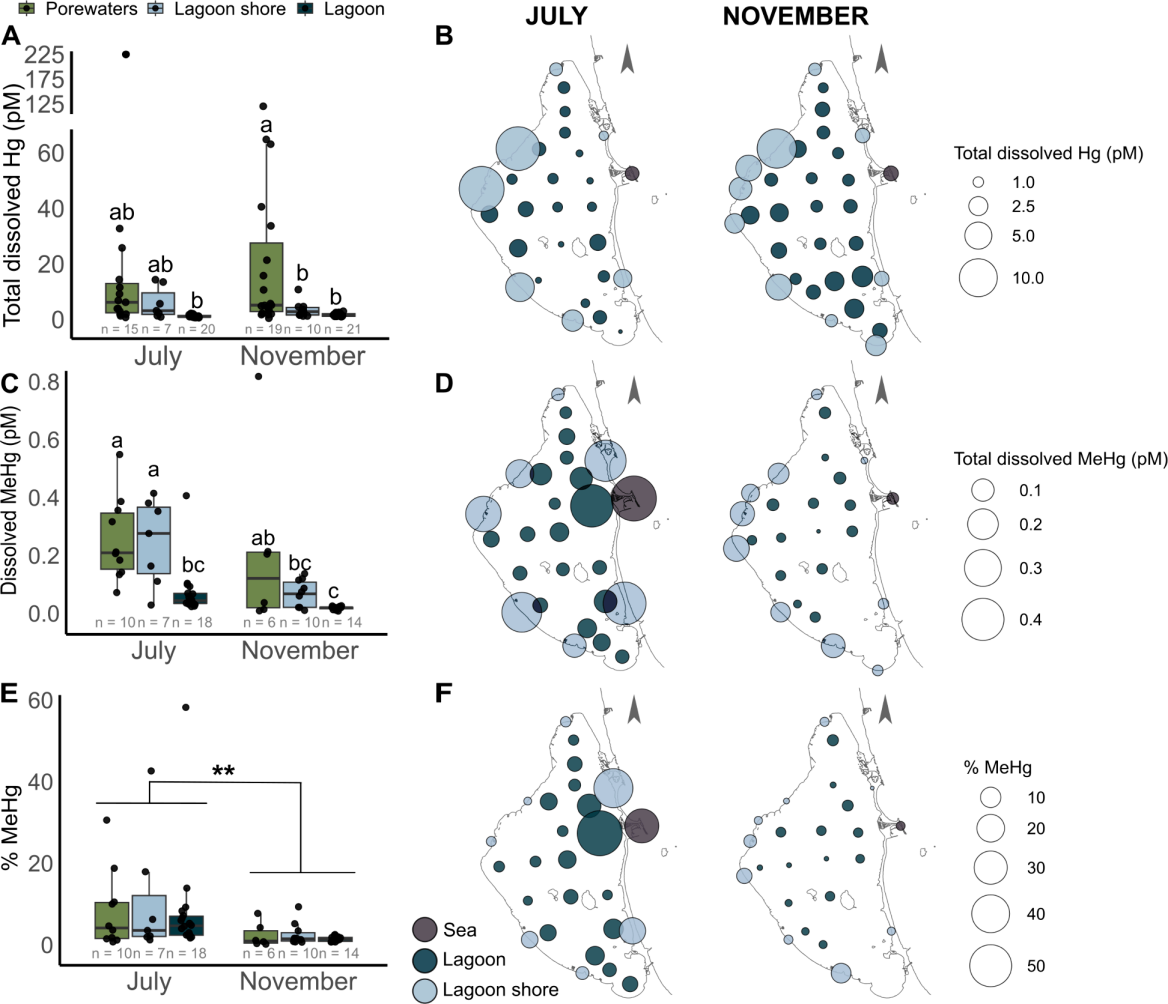
Mercury speciation in surficial and porewaters from the Mar Menor lagoon in two contrasted periods. A, C, and E) Total dissolved Hg and MeHg concentrations expressed in pM or % MeHg compared to total dissolved Hg in the different types of water studied. Significant differences were tested using the posthoc Tukey–Kramer test after removal of the outlier sample PZ4-T1-90. When no significant differences were found among the water types, a sampling period effect was tested using an unbalanced type III two-way ANOVA (*F* = 8.078, *P* < 0.01). For clarity in this boxplot, the two seawater samples from the sea are not represented. B, D, and F) Spatial distribution of total dissolved Hg and MeHg concentrations in pM or % of MeHg compared to the total dissolved Hg in surficial waters in July (left panel) and November (right panel).

Radium isotopes are a powerful tool to successfully track SGD, helping to identify sources and pathways of SGD. Our results reaffirm their easy, accurate, and effective application for a wide range of objectives, such as monitoring Hg inputs and pollution sources. As an alternative, Hg isotopes can also be considered in future research to confirm the source of Hg reaching coastal lagoons. We suggest that combining multiple tools such as radium and Hg isotopes should improve our understanding of these overlooked systems. This approach allows for cross-validation of results and could provide a complementary view of the environmental processes involved.

### Porewater and Lagoon Shore Waters Are Enriched in MeHg

The dissolved MeHg concentrations varied from 0.008 to 0.818 pM and were 4 to 7 times higher in both porewaters and lagoon shore waters relative to lagoon waters (Figure 3B), generating a decreasing gradient along the terrestrial-marine continuum as confirmed by the correlation with ^224^Ra, one of the SGD proxies (ρ = 0.58, *P* < 0.05, Figure S5). No significant correlation was observed with PCA1.

The percentage of MeHg compared to total dissolved Hg (% MeHg), often used as a proxy of net MeHg formation,^69^ reached a median value of 2.2 ± 1.2% ranging from 0.2 to 58.2%. The % MeHg correlated with the ^224^Ra SGD proxy (*P* = 0.035; Figure S4E). We observed a seasonal pattern in % MeHg with higher values in July (median = 4.6 ± 2.1%) compared to November (median = 1.3 ± 0.4%; type III two-way ANOVA, *F* = 8.078, *P* < 0.05; Figure 3E).

It can be hypothesized that groundwater input into the dynamic coastal zone of the Mar Menor lagoon may generate suitable conditions for the biological MeHg formation. The % MeHg was significantly negatively correlated with dissolved O_2_ in the water (*R* ^2^ = −0.32, *P* < 0.05). In addition, we showed that total dissolved Hg was released to the shore through O_2_-depleted SGD associated with DOM enriched in labile protein-like but also in labile humic-like substances (as indicated by a lower FDOM A/C peak ratio). MeHg production is a bio-physicochemical conundrum^70^ influenced by a complex interplay of biological, physical, and chemical factors in which organic matter seems to have a key role. Previous studies showed MeHg formation was enhanced by internally produced labile OM in lakes,^71^ by labile humic-like substances in beaver ponds^15^ and by fresh terrestrial DOM in estuaries.^72^ Moreover, at the sediment-water interface, reductive conditions and sulfide formation^73^ favor the mobility of Hg and MeHg from the sediment to the overlying seawater, where detectable MeHg formation rates have been detected.^74,75^ Hence, in July in Mar Menor, the O_2_-depleted groundwaters from the aquifer and the lagoon shore waters exhibited favorable conditions for putative MeHg formation. Lagoon shore waters enriched in nutrients released from SGD and characterized by high microbial activity (Figure S3D) fueled by reactive natural OM seem to create ideal conditions for MeHg formation, thus representing a potential hotspot for MeHg formation in Mar Menor.

### Characteristics of Different Hg Species in Groundwaters

As a result of mixing processes into the aquifer, the speciation of Hg in porewaters did not exhibit specific spatial or vertical patterns (Table S4). Within the set of samples, the endmembers were selected as described in the methods section for the evaluation of the total dissolved Hg and MeHg fluxes from the different SGD components. Total dissolved Hg concentrations were higher in the meteoric groundwater endmember, whereas MeHg values were greater in the endmember representing the long-scale recirculation of lagoon waters (Table 1). The concentrations used as endmember for the short-scale porewater exchange fluxes were minimal and were also more uncertain for both total dissolved Hg and dissolved MeHg.

**Table 1:** Ancillary Parameters and Hg/MeHg Concentrations from Endmembers Considered for the Flux Calculation from Every SGD Component^a^

	Long-scale lagoon water recirculation			Short-scale porewater exchange			Mneteoric groundwater discharge		
	*Q*_L_			*Q*_S_			*Q*_F_		
	median	min	max	median	min	max	median	min	max
Total dissolved Hg (pM)	3.17	1.15	6.22	2.04	0.00	22.05	4.28	1.71	6.82
Dissolved MeHg (pM)	0.20	0.01	0.14	0.10	0.01	0.59	0.14	0.01	0.32
Salinity (psu)	36.4	29.5	43.0	-	-	-	9.2	9.2	9.3
Dissolved oxygen (mg L^–1^)	1.3	1.2	5.1	-	-	-	5.6	4.7	6.5
pH	7.8	7.1	8.0	-	-	-	7.7	7.5	8.0
ORP (mV)	–157	–261	–78	-	-	-	76	74	78
NO_*x*_ (μM)	1.4	0.6	3.5	-	-	-	831.0	425.1	1236.9
NH_4_^+^ (μM)	24.4	16.2	34.9	-	-	-	5.7	1.4	10.0

a *Q*_F_ is the flow associated with the meteoric groundwater discharge, *Q*_L_ is the long-scale recirculation of lagoon water, and the *Q*_S_ is the short-scale porewater exchange.

### Long-Scale Lagoon Water Recirculation: The Largest Source of Total Dissolved Hg and MeHg

Previous studies have described SGD-driven Hg fluxes.^25–34^ However, the relevance of the different SGD components has not been explored so far. Here, we report total dissolved Hg and MeHg fluxes from different SGD components to a coastal lagoon. The quantification of these SGD components was achieved by integrating radiotracer techniques with hydrogeological modeling. As previously published,^50^ estimated water flows range from 1.7 to 1.9 × 10^4^ m^3^ day^–1^ for meteoric groundwater discharge (*Q*_F_), 2 to 2.3 × 10^6^ m^3^ day^–1^ for long-scale lagoon water recirculation (*Q*_L_), and 0.59 to 4.1 × 10^6^ m^3^ day^–1^ for short-scale porewater exchange (*Q*_S_) (Figure 4). This combined approach is further applied in this study to quantify total dissolved Hg and MeHg fluxes driven by SGD.

**Figure 4:**
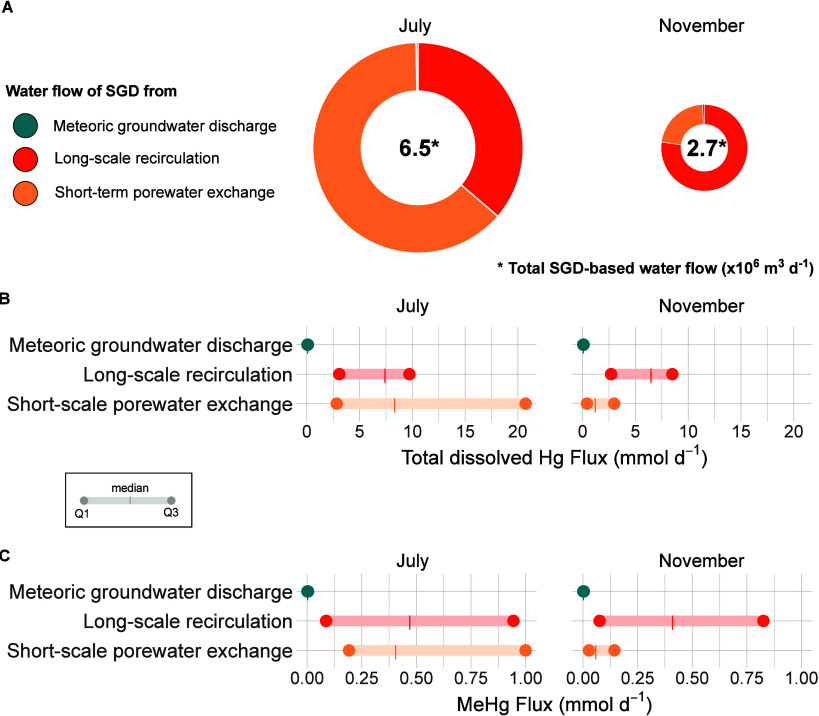
Dissolved total Hg and MeHg fluxes through SGD to the Mar Menor lagoon. A) Total annual water flows from SGD are shown for each sampling period, and proportions of each SGD component are represented as a pie chart. The size of the pie chart is proportional to the total water flow, also indicated within the circle. B–C) Interquartile ranges and median of the SGD-driven total dissolved Hg fluxes (B) and MeHg fluxes (C) to the Mar Menor lagoon for each sampling period. Colors correspond to the three different SGD components.

The total annual SGD-driven total dissolved Hg flux to the Mar Menor lagoon was 4300 mmol year^–1^ (calculated as the sum of the three SGD components). By comparison, the input from the sole permanent stream was trivial, with 60 mmol year^–1^ (considering a water flow of 0.17 m^3^ s^–1^ estimated by Rodriguez-Puig et al.^50^ and a measured total dissolved Hg concentration of 10.48 pM) and the atmospheric inputs rose 4500 mmol year^–1^ (calculated from Cossa et al.^76^). The permanent stream may be a source of total particulate Hg associated with the sediments, not explored in this study but important to consider in the future. Among the different SGD components, *Q*_F_ total dissolved Hg fluxes were negligible compared to the other fluxes representing only 0.5–0.9% of the total. In July, the *Q*_L_ total dissolved Hg fluxes accounted for 82% of the total SGD-based fluxes. In November, 56% of the total SGD-driven total dissolved Hg fluxes originated from *Q*_S_ certainly attributable to the large *Q*_S_ water flows occurring in the summer (Figure 4). The difference on the relative contributions of total dissolved Hg sources is mainly related to the temporal variability of short-scale recirculation, which is driven by temporally variable physical forces (e.g., wave pumping, shear flow, bioturbation).^77^ In the case of Mar Menor, short-scale recirculation increases in summer, most likely because of the increase of bioturbation in summer months,^54^ explaining the large difference of *Q*_s_-driven total dissolved Hg flux between July and November. In line with previous SGD studies as a source of nutrients,^4^ our results confirm the importance of estimating the different components of SGD to obtain an appropriate understanding of their role in coastal areas.

For comparison purposes, the estimated SGD-driven Hg fluxes were normalized by the total area of the semiclosed lagoon (data available in Table S4). The Hg fluxes from SGD to the Mar Menor fell within the lower part of the global reported values, being slightly higher than the one reported in the Bay of Puck^32^ for total Hg (Figure 5 and Figure S6). Despite being low, the reported fluxes may be key and are relevant for the Hg budget in coastal lagoons, particularly in the arid Mediterranean zones threatened by anthropogenic contamination and water scarcity.^78^ In fact, the SGD-derived Hg inventory/stock in the Mar Menor lagoon varied from 920 to 2300 mmol, representing 90 to 249% of the excess Hg inventory estimated by spatial integration in the lagoon.

**Figure 5:**
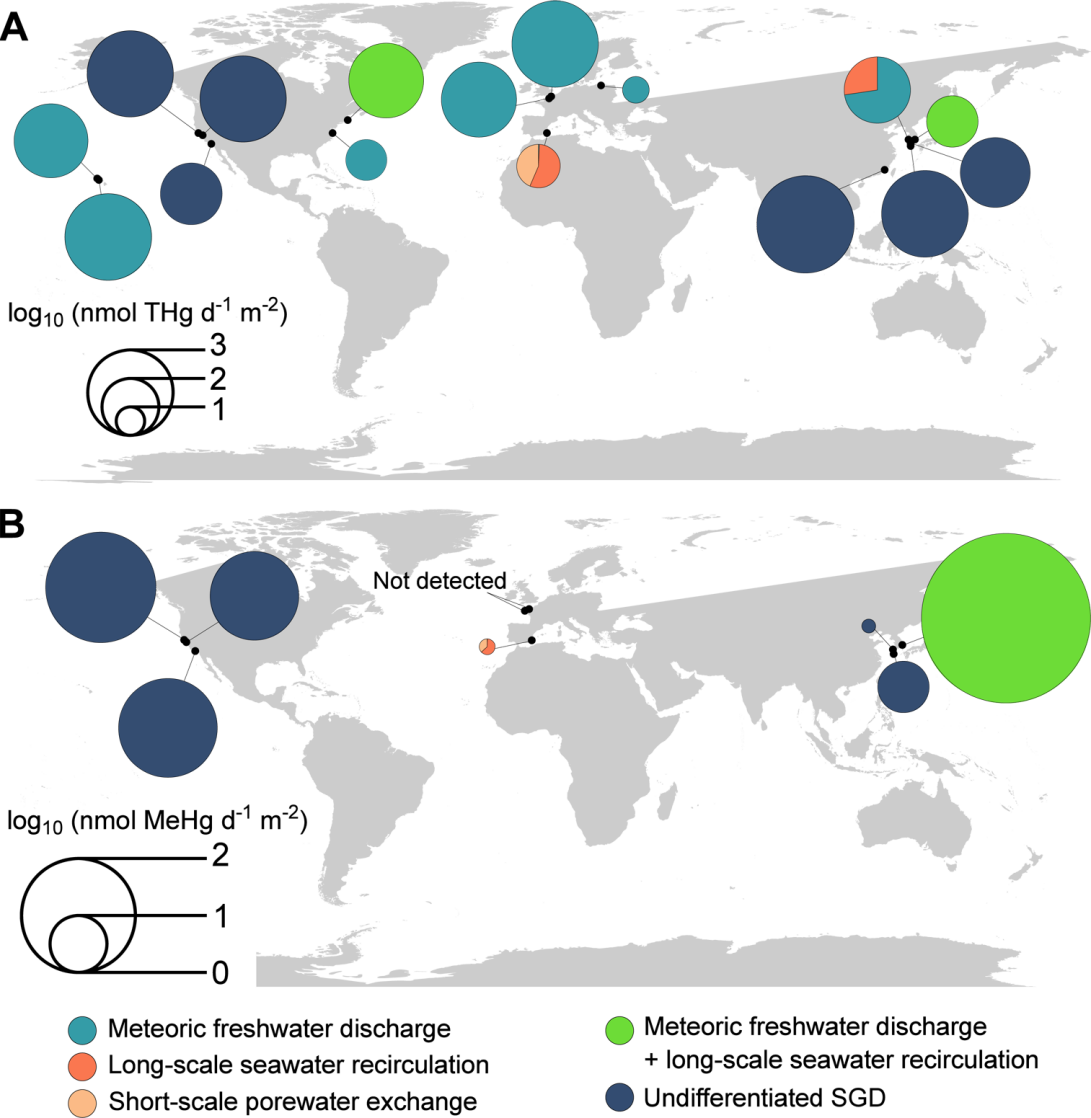
Total Hg and MeHg SGD-driven fluxes to coastal areas reported by the existing studies and the current study. In cases where the fluxes were reported at different periods of the year, an annual average is shown.

To date, MeHg fluxes from SGD have only been described in 8 sites globally^25,26,28,29,33^ (Figure 5 and Figure S7). These investigations suggest that SGD-driven MeHg fluxes may be relevant for the Hg budget at least at local scales. For example, Kim et al.^25^ reported a high MeHg flux from SGD representing 53% of the total Hg input contribution to the Masan Bay (Korea). The SGD-driven annual MeHg fluxes to the Mar Menor lagoon reached a total of 245 mmol of year^–1^. This corresponded to 1.8 nmol year^–1^ m^–2^, 1 order of magnitude lower than the fluxes reported in the Hwasun and Bangdu Bay.^29^ As seen for the total dissolved Hg fluxes, in Mar Menor, meteoric groundwater (*Q*_F_) represented the smallest MeHg input in both sampling periods (i.e., median = 2.7 and 2.5 μmol day^–1^ in July and November, respectively). The *Q*_s_ flux of MeHg reached a median of 405 μmol day^–1^ in July, while in November this flux was lower with a median of 59 μmol day^–1^ (Figure 4C). Although the larger SGD-driven MeHg input to the Mar Menor lagoon was the *Q*_L_ flux in both seasons, with values ranging from 410 to 944 μmol day^–1^ (Figure 4C), the short-scale porewater exchange (*Q*_s_) was stronger in July, reaching the range of the *Q*_L_ flux. The SGD-derived inventory of MeHg ranged from 56 to 128 mmol, representing 226–336% of the MeHg inventory in the lagoon. Our study thus discloses the relevance of SGD as an input of both Hg and MeHg to the coastal lagoon.

At a global scale, 60 to 70% of Hg is originated from re-emission of Hg mainly emitted during the industrial era.^79^ Our results indicate that this legacy Hg stored in the sediments is remobilized through long-scale recirculation (median annual fluxes total dissolved Hg = 2500 mmol year^–1^ and MeHg = 160 mmol year^–1^) and represents one of the main sources of Hg to the Mar Menor lagoon throughout the year after atmospheric deposition. This novel finding is particularly relevant considering that most of the studies only report fluxes from meteoric groundwater discharge^26,27,32,34^ (Figure 5; Figures S6–S7), resulting in a large underestimation of the relevance of SGD as an input of nutrients, OM, or contaminants such as metal to the coastal zones. Thus, we call for a global effort to assess Hg inputs from SGD-driven fluxes and to pay special attention to legacy Hg stored in sediments, which represents a significant threat to the effectiveness of the Minamata Convention aimed at reducing Hg levels. Further investigations may also evaluate the risks of MeHg formation in these dynamically sensitive transitional zones.

## Supplementary Material



## Notes

The Supporting Information is available free of charge at https://pubs.acs.org/doi/10.1021/acs.est.5c07191. The complete dataset can be downloaded at https://doi.org/10.5281/zenodo.17045205.

Detailed description of additional methods, supporting tables with detailed data used for calculation of the Hg and MeHg fluxes and extra figures showing specific patterns of the ancillary parameters, and comparing the reported Hg and MeHg fluxes with the literature (PDF)

## ○

J.G.-O. died on July 5, 2022.
