# Supplementary material for: Unveiling Hidden Mercury and Methylmercury Sources: The Role of Submarine Groundwater Discharge in Coastal Lagoons
Source: Environ Sci Technol. 2025 Sep 15;59(38):20653–64. doi: 10.1021/acs.est.5c07191 (PMC12489978; doi:10.1021/acs.est.5c07191)
Supplement: Supplementary file 1 [file es5c07191_si_001.pdf]

Supplementary Information

Unveiling hidden mercury and methylmercury sources: the role of submarine groundwater discharge in coastal lagoons

Céline Lavergne, Júlia Rodriguez-Puig, Clara Ruiz-González, María Montero-Curiel, Gemma Casas, Daniel Romano-Gude, Irene Alorda-Montiel, Júlia Dordal-Soriano, Aaron Alorda-Kleinglass, Marc Diego-Feliu, Javier Gilabert, Alex Campillo-de la Maza, Cristina Romera-Castillo, Natalia Torres-Rodriguez, Lars-Eric Heimbürger-Boavida, Jordi García-Orellana, Valentí Rodellas, Andrea G. Bravo

Corresponding authors: Céline Lavergne (lavergne@icm.csic.es) and Andrea G. Bravo (andrea.bravo@icm.csic.es)

Index

Supplementary Methods 1. *Analytical methods for mercury (Hg) and methylmercury (MeHg) measurements 3*
Supplementary Methods 2. *Analytical methods of ancillary parameters 4*
Supplementary Methods 3. *Calculation method of SGD-derived Hg inventory and excess inventory of Hg in the lagoon 5*

Table S1. *Sampling methods for water samples to measure total dissolved Hg and dissolved MeHg 6*
Table S2. *Concentrations of total dissolved (dHg) and dissolved MeHg (dMeHg) expressed in pM and statistical associated values as well as minimum and maximum salinity. 6*
Table S 3. *Literature review of the total Hg partitioning coefficient (K_D) from sites with similar characteristics compare to the studied coastal site. * median-based K_D from multiple data 7*
Table S 4. *Endmembers considered for the flux calculation from every SGD component: Q_F is the flow associated with the meteoric discharge of fresh groundwater, Q_L is the long-scale recirculation of lagoon water, and the Q_S is the short-scale porewater exchange. ....* ***Error! Bookmark not defined.***
Table S5. *Total dissolved Hg and dissolved MeHg fluxes through SGDs to the Mar Menor lagoon expressed in different units. The total study area of 135 km^2 was considered for area normalization. Water flow values are detailed as well as the fluxes expressed in different units for comparison purpose. The endmembers are given in the Table S 3. .... 10*

Figure S 1. *Main patterns of the geochemical and biological parameters. Boxplots showing the variations of 4 parameters according to the water type (porewaters, superficial lagoon shore waters and superficial lagoon waters) and the sampling period (July and November). 11*
Figure S 2. *Boxplots showing the variations of 6 geochemical features according to the water type (porewaters, lagoon shore waters and lagoon waters) and the sampling period (July and November). Significant differences were tested using post-hoc Tukey-Kramer test after unbalanced type III two-way ANOVA and indicated by the letters. For clarity in this boxplot, the two seawater samples from the sea are not represented. 12*
Figure S 3. *Main patterns of the geochemical and biological parameters. Boxplots showing the variations of 4 geochemical and biological features according to the water type (porewaters, superficial lagoon shore waters and superficial lagoon waters) and the sampling period (July and November) which is displayed by colors. Significant differences were tested using post-hoc Tukey-Kramer test after unbalanced type III two-way ANOVA and indicated by the letters. Note that in panel A, no significant differences were detected among the*

tested conditions. FDOM stands for fluorescent dissolved organic matter. The reader may refer to the online version for colors. 13
Figure S 4. *Non-parametric correlations of Hg species and 224-Ra or PCA components in both sampling periods. A-B) Non-parametric correlations of dissolved total Hg (THg) with the concentration of ^{224}Ra and the first PCA component coordinates and associated statistics. C-D) Non-parametric correlations of dissolved methylmercury (MeHg) with the concentration of ^{224}Ra and the first PCA component coordinates and associated statistics. E-F) Non-parametric correlations of the % of dMeHg compared to dHg with the concentration of ^{224}Ra and the second PCA component coordinates and associated statistics.* 14
Figure S 5. *Linear regressions of total dissolved Hg and SGD proxies (top panel: 224Ra, bottom panel: first component of PCA) separated by sampling periods.* 15
Figure S6. *SGDs-based THg fluxes worldwide. The fluxes are reported in nmol d^{-1} m^{-2} and separated by SGD component when available (if not, the category "undifferentiated SGD" was used).* 16
Figure S7. *SGDs-based MeHg fluxes worldwide. The fluxes are reported in pmol d^{-1} m^{-2} and separated by SGD components when available.* 17

Supplementary Methods 1. Analytical methods for mercury (Hg) and methylmercury (MeHg) measurements

Dissolved total mercury (dHg) and methylmercury (dMeHg) concentrations in water samples

All glass vials were acid-washed, burnt, and rinsed 3 times with the sample on site before sampling. Water was collected into 60 mL borosilicate vials for THg and 250 mL amber borosilicate bottles for MeHg and acidified to 0.4 % v/v (HCl Ultrex II, J. T. Baker, USA) on site. Samples were stored in the dark at 4 °C until analysis.

Dissolved total Hg. Dissolved total Hg (dHg) was measured following a modified version of the USEPA1631 method (US EPA, Method 1631, 1999) based on cold vapor atomic fluorescence spectrometry (CV-AFS, Brooks Rand Model III, USA) coupled to a custom made semi-automatic single gold trap[1]. The BrCl solution was prepared with a double-distilled HCl, and potassium bromide (Sigma Aldrich, USA) and potassium bromate (Sigma Aldrich, USA) that were pre-heated during 4h at 250°C to remove traces of Hg. The absence of contamination in the reagents was verified for each new batch by repetitive additions[2]. The ORMS-5 and NIST-3133 (National Institute of Standards and Technology) were used as certified material achieving 101 ± 6 % and 98 ± 7 % of recovery, respectively. The calibration curves ($R^2 > 0.997$) were performed by spiking NIST-3133 into purged Hg-free ultrapure water (18 MΩ cm, MilliQ). The limit of detection was 0.03 pM.

Dissolved MeHg. Dissolved MeHg (dMeHg) was measured by species specific isotopic dilution gas chromatography coupled to a sector field ICP-MS (SS-GC-SF-ICP-MS). As previously described in Heimbürger et al.[1], filtered water samples were buffered to pH 3.9 using a sodium acetate buffer solution (ULTREX II Ultrapure Reagent, J.T. Baker) and spiked with solutions enriched in specific isotopes: 0.08-2.24 pg g^{-1} $i^{199}Hg$ and 0.01-4.52 pg g^{-1} $Me^{201}Hg$ to ensure a robust quantification with optimal excess ratios[2]. The exact spike concentrations were assessed by reverse isotope dilution using the NIST1641E certified material [2]. Derivatization was performed with the addition of 5% v/v solution of sodium tetrapropylborate (Merseburger Spezialchemikalien) and 190-200 µL of isooctane (Sigma-Aldrich) and an agitation of 15 min on an orbital shaker (Edmund Buhler KS15). MeHg and inorganic Hg species in the resulting organic phase were then measured by GC-SF-ICP-MS (GC, THERMO GC 1300 with GC220 transfer module coupled to Thermo Element XR). Detection limit was 0.002 pM.

Particulate total Hg concentrations in sediments

Alorda et al.,[3] reported THg concentrations of a total of 12 sediment cores (120 cm long, 8.5 cm internal diameter) collected in December 2020 by divers and sliced every centimeter in the laboratory within the following 12 h. Briefly, sediment samples were dried at 65 °C, grounded with an agate mortar and pestle and particulate total Hg (pHg) was measured via atomic absorption spectrometry (AMA254, LECO). Accuracy was validated using the certified reference material MESS-3 (National Water Research Institute, Canada). The mean concentrations used for this study were 139.9 ng THg g^{-1} dry sediment.

Supplementary Methods 2. Analytical methods of ancillary parameters

Physicochemical parameters

Physicochemical parameters (i.e. temperature, pH, dissolved oxygen – DO - concentration, conductivity, salinity and oxidation/reduction potential - ORP) were measured *in situ* using a multiparametric probe (YSI Professional Plus). Inorganic nutrient concentrations (i.e., nitrate (NO_2^-) and nitrite (NO_3^-), phosphate (PO_4^{2-}) and ammonium (NH_4^+)) were assessed using an AA3 HR autoanalyser (Seal Analytical). Dissolved organic carbon (DOC) was measured in filtered acidified water samples (orthophosphoric acid; pH <2) according to Romera-Castillo et al.[4] using a Shimatzu TOC-V carbon analyzer and based on the methodology described by Álvarez-Salgado et al.[5]. The fluorescent dissolved organic matter (FDOM) was studied in water filtered through pre-combusted GF/F filters and stored a 4 °C until analysis. The fluorescence emission excitation matrices (EEMs) were acquired from the filtered water samples using LS 55 Luminescence spectrometer (Perkin Elmer), corrected for blank value, instrument drift, Raman scattering and inner filter effect with the drEEM toolbox for MATLAB version 9.13[6] and the peaks were expressed as Raman Unit (RU). The quality and composition of FDOM was assessed by the ratios of the different peaks (i.e. A, T, C, M and B).

Water samples were collected to analyze ^{224}Ra, ^{226}Ra, and ^{228}Ra, which are commonly used as a tracers of SGD components (including groundwater discharge, long- and porewater exchange)[7,8]. A description of Ra analytical procedures and data for these samplings is provided in Rodellas et al.[56].

Prokaryotic heterotrophic activity

Bulk prokaryotic heterotrophic activity from porewaters and lagoon water was measured using the ^{3}H-leucine incorporation method[9] with the modifications of Smith and Azam[10]. Samples were incubated 2-4 h in the dark at *in situ* temperature with ^{3}H-labelled leucine (160 Ci mmol, 20 nM final conc.)[10]. Leucine incorporation into protein was determined by precipitation with TCA (5 % final conc.) and centrifugation, followed by liquid scintillation counting (Beckman). Bulk prokaryotic activity was expressed as pmol Leu L^{-1} h^{-1}.

Supplementary Methods 3. Calculation method of SGD-derived Hg inventory and excess inventory of Hg in the lagoon

To estimate the relevance of SGD inputs into the lagoon, the SGD-derived Hg inventory was calculated and contrasted to the inventory of Hg in the lagoon according to Rodellas et al.[11] (detailed in Supporting Information S2) First, the SGD-derived Hg inventory (mol) was the estimated Hg flux from SGD (mol d^{-1}) multiplied by the residence time of the water into the lagoon (days). The inventory of Hg (mol) corresponded to the values of Hg concentrations in the lagoon waters were spatially interpolated (IDW Interpolation, QGIS; output raster size with 1000 rows) multiplied by the depth at each site (spatially integrated with QGIS) and the total area of the lagoon (135 km^2). By diving the SGD-derived Hg inventory by the inventory of Hg, we obtained an estimate of the relevance of the SGD for Hg input being a value >1 considered relevant for the Hg inventory.

Table S1. Sampling methods for water samples to measure total dissolved Hg and dissolved MeHg

<table>
<tr><th>Sample type</th><th>Water type</th><th>Collection method</th><th>Filtration method</th><th>Hg specie measured</th></tr>
<tr><td>POREWATERS</td><td>Groundwater (10 transects of three sites from terrestrial to marine: T,O and M)</td><td>PTFE-based piezometers coupled to Masterflex™ L/S™ PTFE-Diaphragm Pump System</td><td rowspan="3">PFA Tefzel® Clamp in which a sequential filtration was performed using pre-burnt GF/D (2.7 μm), GF-A (1.6 μm) and GF/F (0.7 μm) glass fiber filters</td><td rowspan="3">Total dissolved Hg and dissolved MeHg (fixed with 0.4 % v/v HCl Ultrex II, J. T. Baker, USA)</td></tr>
<tr><td>LAGOON SHORE</td><td>Surficial water</td><td rowspan="2">PTFE tubing connected to the Masterflex™ L/S™ PTFE head pump</td></tr>
<tr><td>LAGOON</td><td>Surficial water</td></tr>
</table>

Table S2. Concentrations of total dissolved (Hg) and dissolved MeHg (MeHg) expressed in pM and statistical associated values as well as minimum and maximum salinity.

	Lagoon water	Fresh coastal porewater	Brackish coastal porewater	Saline coastal porewater	Lagoon surficial porewater (estimated)
**Dissolved Hg (pM)**					
IQR	0.68	2.00	30.80	3.52	2.60
Median	1.17	4.28	12.93	4.34	3.49
Q1	1.00	3.41	2.16	2.31	2.11
Q3	1.67	5.41	32.96	5.83	4.72
Salinity min	41.1	2.6	10.2	29.5	NA
Salinity max	42.9	9.3	25.4	43.0	NA
**Dissolved MeHg (pM)**					
IQR	0.03	0.15	0.03	0.37	0.10
Median	0.03	0.14	0.21	0.23	0.14
Q1	0.02	0.08	0.18	0.06	0.08
Q3	0.02	0.23	0.21	0.43	0.19
Salinity min	41.1	8.3	10.2	29.5	NA
Salinity max	42.8	9.3	25.0	43.0	NA

Table S 3. Literature review of the total Hg partitioning coefficient (K_D) from sites with similar characteristics compare to the studied coastal site. * median-based K_D from multiple data

Reference	Site	Type of system	Salinity	K_D (L kg^{-1})
Cossa and Martin (1991) [12]	Rhône (France)	Coastal	Brackish/Saline	2.5 x10^5
Balls (1989) [13]	Belgian coast, Bristish estuaries	Coastal	Brackish/Saline	3.2 x10^5
Coquery and Cossa (1995)* [14]	North Sea	Coastal	Saline	4.4 x10^5
Coquery and Cossa (1995)* [14]	North Sea	Coastal	Brackish	5.1 x10^5
Coquery and Cossa (1995)* [14]	Elbe (Germany)	Coastal	Brackish	4.1 x10^5
Coquery et al. (1995) [15]	Lena (Siberia)	Coastal	Brackish/Saline	3.2 x10^5
Coquery et al. (1995) [15]	Ob (siberia)	Coastal	Brackish/Saline	1.6 x10^5
Coquery et al. (1995) [15]	Yenisei (Siberia)	Coastal	Brackish/Saline	1.6 x10^5
Stordal et al. (1996) [16]	Texas estuaries (USA)	Coastal	Brackish/Saline	7.9 x10^4
Coquery et al. (1997) [17]	Loire (France)	Coastal	Fresh to saline	4.0 x10^5
Coquery et al. (1997) [17]	Seine (France)	Coastal	Fresh to saline	7.9 x10^5
Muresan et al. (2007) [18]	Thau Lagoon (France)	Coastal lagoon	Saline	6.2 x10^4
Bloom et al. (2004) [19]	Venice Lagoon (Italy)	Coastal lagoon	-	4.4 x10^5
Allison & Allison US EPA (2005)* [20]	-	-	-	7.9 x10^4
Covelli et al. (2011) [21]	Ravenna Lagoon (Italy)	Coastal lagoon	Saline	1.9 x10^6
Covelli et al. (2011) [21]	Grado Lagoon (Italy)	Coastal lagoon	Saline	2.0 x10^5
Emili et al. (2012) [22]	Marano Lagoon (Italy)	Coastal lagoon	Brackish	1.7 x10^5
Emili et al. (2012) [22]	Marano Lagoon (Italy)	Coastal lagoon	Brackish	1.2 x10^5
Emili et al. (2012) [22]	Marano Lagoon (Italy)	Coastal lagoon	Brackish	3.7 x10^3
Emili et al. (2012) [22]	Marano Lagoon (Italy)	Coastal lagoon	Brackish	4.2 x10^4
Emili et al. (2012) [22]	Marano Lagoon (Italy)	Coastal lagoon	Brackish	4.0 x10^5
Emili et al. (2012) [22]	Marano Lagoon (Italy)	Coastal lagoon	Brackish	2.0 x10^5
Emili et al. (2012) [22]	Marano Lagoon (Italy)	Coastal lagoon	Brackish	6.2 x10^4
Emili et al. (2012) [22]	Marano Lagoon (Italy)	Coastal lagoon	Brackish	1.7 x10^5
Bratkic et al. (2013) [23]	Gulf of Trieste (Slovenia)	Coastal	Brackish/Saline	4.2 x10^4
Bratkic et al. (2013) [23]	Gulf of Trieste (Slovenia)	Coastal	Brackish/Saline	5.1 x10^6
Oliveri et al. (2016) [24]	Augusta Bay (Italy)	Coastal lagoon	-	6.3 x10^1
Oliveri et al. (2016) [24]	Augusta Bay (Italy)	Coastal lagoon	-	3.2 x10^2
Oliveri et al. (2016) [24]	Augusta Bay (Italy)	Coastal lagoon	-	6.3 x10^4
Oliveri et al. (2016) [24]	Augusta Bay (Italy)	Coastal lagoon	-	6.3 x10^5
Emili et al. (2016) [25]	Mar Piccolo (Italy)	Coastal lagoon	Brackish	2.5 x10^5
Emili et al. (2016) [25]	Mar Piccolo (Italy)	Coastal lagoon	Brackish	2.0 x10^5
Cesario et al. (2017) [26]	Tagus estuary (Portugal)	Coastal	Brackish	5.0 x10^5
Cesario et al. (2017) [26]	Tagus estuary (Portugal)	Coastal	Brackish	1.6 x10^5
Cesario et al. (2017) [26]	Tagus estuary (Portugal)	Coastal	Brackish	1.6 x10^5
Cesario et al. (2017) [26]	Tagus estuary (Portugal)	Coastal	Brackish	7.9 x10^4
			**MEDIAN Kd**	**1.8 x10^5**
			**LOG 10 (median Kd)**	**5.266**

Supplementary Information

Table S4. Hg speciation and ancillary parameters from individual groundwater samples (Sampling from July).

Site	Influence	P	Depth (cm)	THg (pM)	MeHg (pM)	%MeHg	Temp (°C)	Salinity (psu)	pH	dO2 (mg L^{-1})	ORP (mV)	NO_3^- (uM)	NH_4^+ (uM)	$PO_4$2- (uM)	DOC (uM)	FDOM T/C ratio	FDOM A/C ratio	Bacterial abundance (cell mL^{-1})	Leucine uptake (pmol Leu L^{-1} h^{-1})
PZ1	Agricul-tural	0	20	32.73	NA	NA	27.1	13.4	6.8	0.46	-295.1	40.6	52.2	4.88	582.87	0.39	1.61	$2.1x10^6$	31.98
		M	15	3.86	0.39	10.0	28.1	32.8	7.1	1.32	-218.0	0.7	21.3	1.03	375.87	0.55	2.02	$2.9x10^5$	3.46
PZ2	Urban	T	50	3.92	0.14	3.6	26.3	8.3	7.7	NA	-82.5	19.5	51.8	0.99	1165.07	0.61	1.89	$1.3x10^5$	2.47
		0	20	9.16	NA	NA	26.4	42.3	7.8	0.56	-273.0	0.4	51.5	3.10	1024.08	0.54	1.74	$5.1x10^5$	5.45
		M	25	6.17	0.07	1.2	27.5	40.0	8.0	1.21	-260.7	2.7	34.9	2.26	348.59	0.61	1.68	$7.9x10^5$	363.19
PZ4	Wetland	T	90	225.25	NA	NA	25.4	9.2	7.6	1.26	-300.0	337.4	107.9	0.86	326.30	0.59	1.44	$1.3x10^5$	1.72
		T	40	14.34	0.21	1.5	28.4	16.6	7.7	2.98	-272.5	35.7	77.4	7.70	857.10	0.69	1.56	$1.5x10^6$	136.60
PZ5	Urban	0	20	2.89	NA	NA	26.5	4.8	7.8	1.50	13.0	1494.0	3.9	0.29	85.22	3.21	2.14	$6.6x10^4$	10.66
PZ6	Mining	T	55	1.27	0.13	10.5	27.6	11.5	7.8	2.96	-120.6	1.3	15.6	0.35	275.72	0.39	1.81	$1.4x10^5$	1.19
		0	20	11.52	0.21	1.8	27.0	25.0	7.8	1.45	-140.4	0.1	46.2	0.42	723.57	0.36	2.01	$1.4x10^5$	0.60
		M	20	0.73	NA	NA	29.1	25.4	7.0	8.07	-162.2	0.7	26.8	0.42	268.91	1.95	1.65	$3.0x10^5$	1.80
PZ7	Mining	T	70	25.75	0.18	0.7	28.7	12.2	7.5	1.65	39.8	0.2	25.6	0.12	203.10	0.37	1.86	$3.5x10^5$	2.16
		0	15	6.82	0.32	4.6	26.1	9.3	8.0	4.70	74.3	395.9	1.4	0.37	140.45	0.73	1.73	$2.7x10^5$	1.00
		M	20	1.90	0.36	18.8	27.2	20.3	7.7	2.22	-159.0	0.2	10.5	0.18	167.18	2.14	1.97	$2.7x10^5$	3.50
PZ10	Urban	0	20	1.79	0.55	30.6	27.8	29.5	7.9	1.26	-78.4	0.5	16.2	0.56	441.71	0.99	1.59	$6.9x10^5$	157.28

Table S4. (continued) Sampling from November.

Site	Influence	P	Depth (cm)	THg (pM)	MeHg (pM)	%MeHg	Temp (°C)	Salinity (psu)	pH	dO2 (mg L^{-1})	ORP (mV)	NO3- (uM)	NH4+ (uM)	PO4 (uM)	DOC (uM)	FDOM T/C ratio	FDOM A/C ratio	Bacterial abundance (cell mL^{-1})	Leucine uptake (pmol Leu L^{-1} h^{-1})
PZ1	Agricul-tural	T	100	64.80	NA	NA	17.1	11.8	7.2	3.03	-82.7	1136.6	2.6	0.42	271.31	0.21	1.73	3.4x10^5	2.47
		0	50	15.74	NA	NA	18.9	16.7	7.1	4.03	-22.8	351.5	1.5	0.19	167.70	0.25	1.81	1.4x10^5	0.84
		M	40	33.68	NA	NA	17.4	16.1	7.2	4.55	33.9	370.3	0.5	0.23	166.09	0.26	1.91	6.8x10^5	3.46
PZ2	Urban	T	50	4.28	NA	NA	17.8	3.6	7.5	5.69	97.9	215.6	0.7	0.33	224.37	0.34	2.12	2.4x10^5	1.12
		0	20	4.82	NA	NA	16.1	31.3	7.5	2.57	-117.0	1.4	201.9	1.35	535.16	0.34	1.82	2.1x10^5	18.73
		0	80	10.62	0.82	7.7	18.3	22.0	7.5	6.08	-195.9	4.4	64.2	0.21	285.65	1.64	1.76	2.8x10^5	0.29
PZ3	Urban	0	30	21.33	NA	NA	15.7	14.6	7.4	3.11	97.0	1387.6	8.7	0.39	178.42	0.38	1.88	1.7x10^5	6.84
PZ4	Wetland	T	50	120.07	NA	NA	17.5	16.1	7.5	3.76	-60.0	1.2	206.9	3.03	2450.30	0.14	1.75	1.0x10^6	74.32
		0	40	40.57	NA	NA	17.2	21.6	7.4	3.30	-185.5	17.7	212.7	7.16	3390.14	0.19	1.82	1.1x10^6	10.35
PZ5	Urban	0	80	5.13	NA	NA	19.9	2.6	7.3	3.90	70.6	1266.8	0.7	0.28	67.76	0.28	1.84	4.7x10^4	0.80
PZ6	Mining	T	60	1.95	NA	NA	17.8	14.3	7.1	3.36	-102.3	2.8	151.2	0.10	235.01	0.35	1.68	1.3x10^5	0.96
		0	70	1.71	NA	NA	17.7	8.7	7.1	3.01	-18.8	0.2	53.9	0.15	139.77	0.29	1.82	5.0x10^4	0.54
		M	40	0.43	NA	NA	17.2	24.8	7.2	5.61	-122.5	5.9	112.0	0.10	181.72	0.29	1.74	8.0x10^4	1.18
PZ7	Mining	T	70	3.41	0.04	1.1	17.7	10.2	7.7	4.22	76.1	72.7	21.7	0.06	145.28	0.45	1.94	8.1x10^5	2.89
		0	60	5.03	0.21	4.2	17.9	10.2	7.8	3.76	63.3	558.5	49.0	0.11	149.69	0.59	1.79	3.3x10^5	43.28
		M	10	5.69	0.01	0.2	17.9	9.2	7.5	6.50	78.3	1232.3	10.0	0.08	56.89	0.98	2.36	3.0x10^5	1.76
PZ8	Urban	T	40	63.10	0.20	0.3	16.2	13.7	7.4	4.60	44.0	413.8	42.0	0.20	717.57	0.30	1.87	7.3x10^5	7.14
PZ9	Urban	0	50	1.78	0.01	0.8	17.6	43.0	7.7	5.13	-96.1	1.8	27.5	0.30	264.89	0.65	2.12	5.2x10^5	11.96
PZ10	Urban	0	50	2.23	NA	NA	18.0	14.4	7.8	4.53	-159.1	1.0	16.7	0.37	149.73	0.43	1.79	3.6x10^5	2.11

Table S5. Total dissolved Hg and dissolved MeHg fluxes through SGDs to the Mar Menor lagoon expressed in different units. The total study area of 135 km^2 was considered for area normalization. Water flow values estimated in Rodriguez-Puig et al.[8] are detailed as well as the fluxes expressed in different units for comparison purpose. The endmembers are given in the main text (Table 1).

			Entire year			July			November		
			Recirculated SGD	PEX SGD	Fresh SGD	Recirculated SGD	PEX SGD	Fresh SGD	Recirculated SGD	PEX SGD	Fresh SGD
**SGD rate**		m^3 d^{-1}	2.16 x10^6	2.31 x10^6	1.78 x10^4	2.34 x10^6	4.09 x10^6	1.89 x10^4	2.04 x10^6	5.90 x10^5	1.72 x10^4
**Total Hg**											
Flux THg	ANNUAL	nmol m^{-2} yr^{-1}	19.0	15.0	0.20						
Flux THg	ANNUAL	g yr^{-1}	510	400	6						
Flux THg	ANNUAL	mol yr^{-1}	2.50	2.00	0.03						
Flux THg	DAILY	ng day^{-1} m^{-2}				11.0	12.4	0.1	9.6	1.8	0.1
Flux THg	DAILY	nmol day^{-1} m^{-2}				0.055	0.062	0.001	0.048	0.009	0.001
**MeHg**											
Flux MeHg	ANNUAL	nmol m^{-2} yr^{-1}	1.19	0.70	0.01						
Flux MeHg	ANNUAL	g yr^{-1}	0.03	0.02	0.00						
Flux MeHg	ANNUAL	mol yr^{-1}	0.16	0.09	0.00						
Flux MeHg	DAILY	pg day^{-1} m^{-2}				750	647	4	656	93	4
Flux MeHg	DAILY	pmol day^{-1} m^{-2}				3.48	3.00	0.02	3.04	0.43	0.02

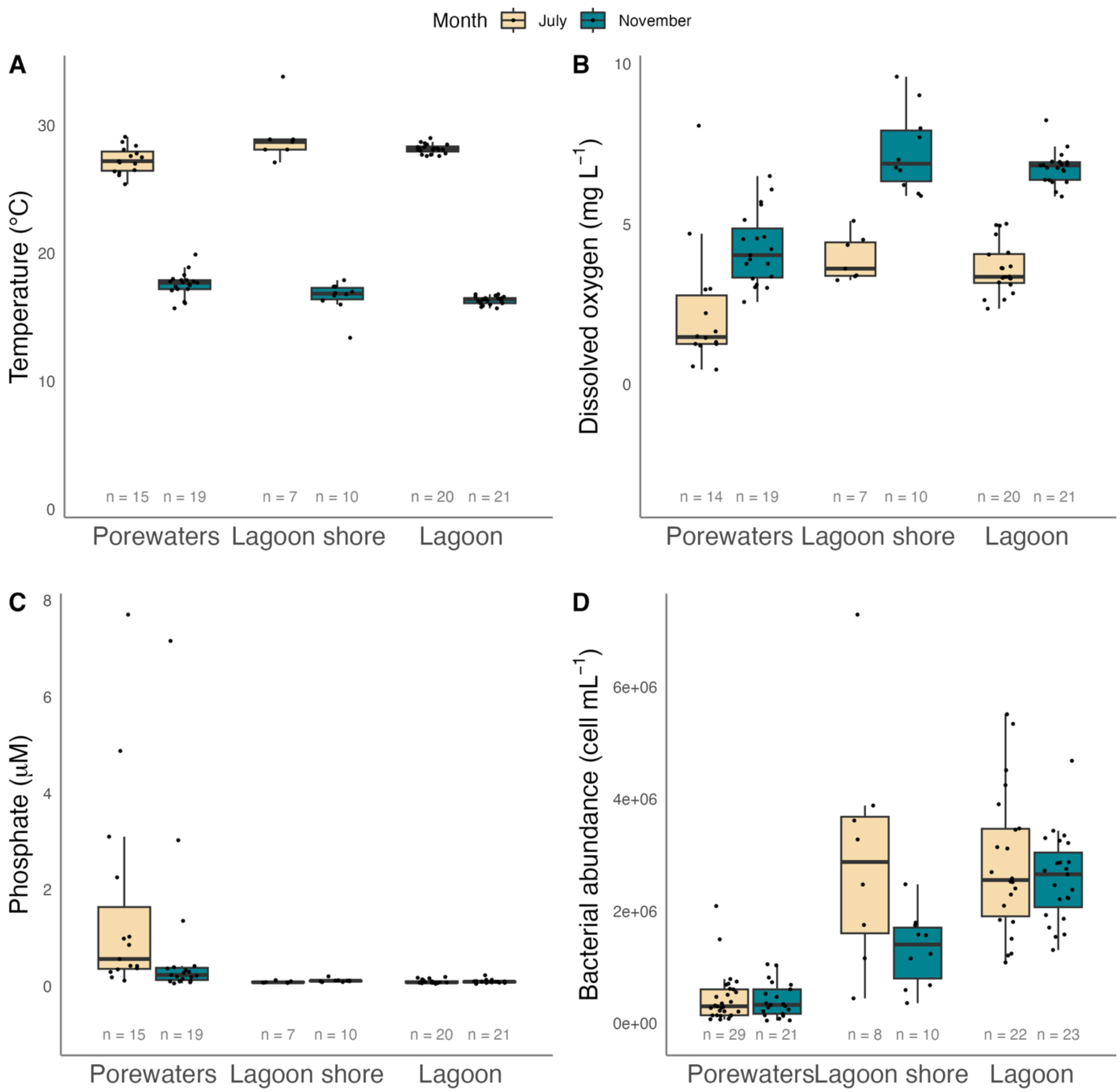

Figure S 1. Main patterns of the geochemical and biological parameters. Boxplots showing the variations of 4 parameters according to the water type (porewaters, superficial lagoon shore waters and superficial lagoon waters) and the sampling period (July and November).

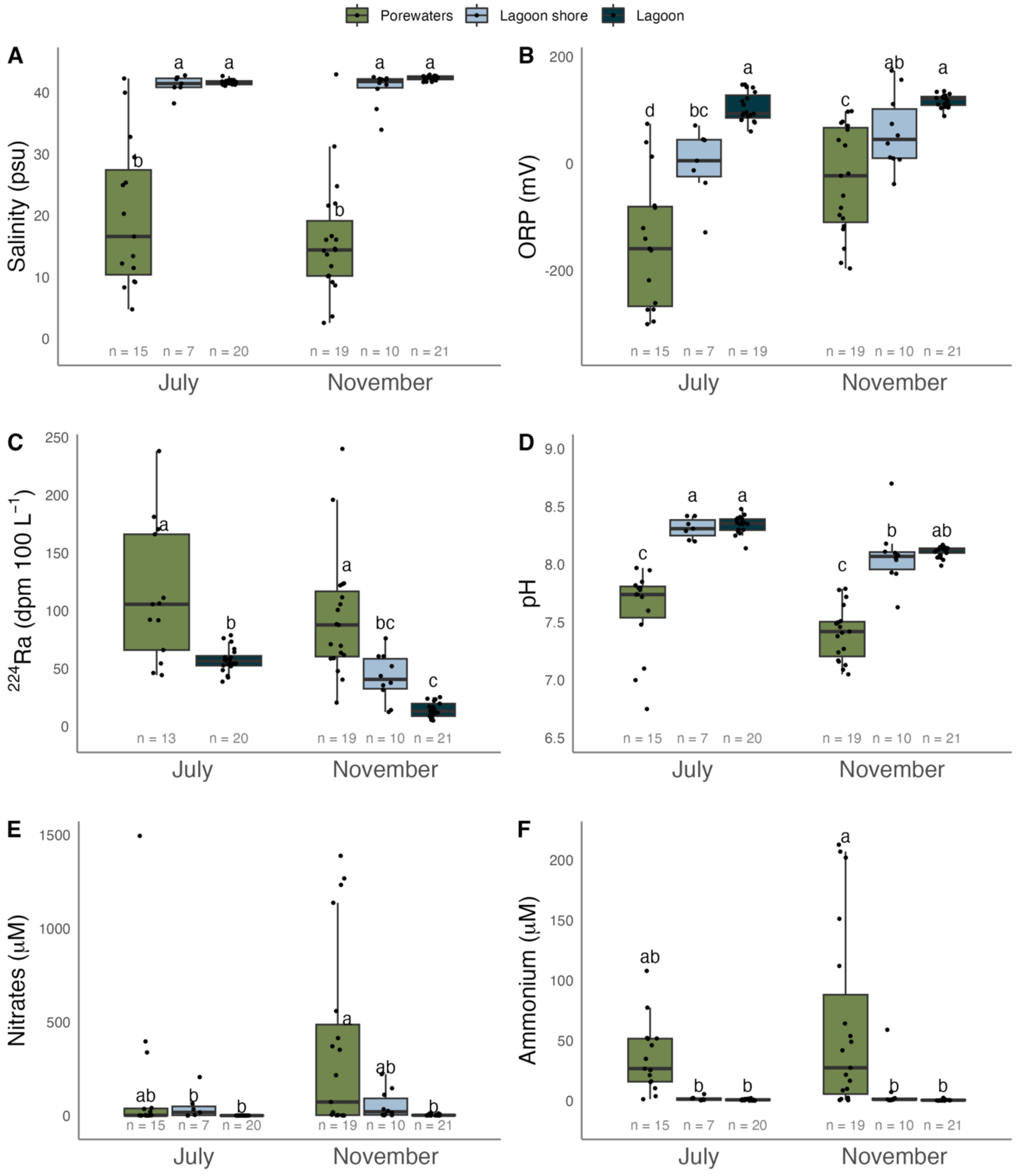

Figure S 2. Boxplots showing the variations of 6 geochemical features according to the water type (porewaters, lagoon shore waters and lagoon waters) and the sampling period (July and November). Significant differences were tested using post-hoc Tukey-Kramer test after unbalanced type III two-way ANOVA and indicated by the letters. For clarity in this boxplot, the two seawater samples from the sea are not represented.

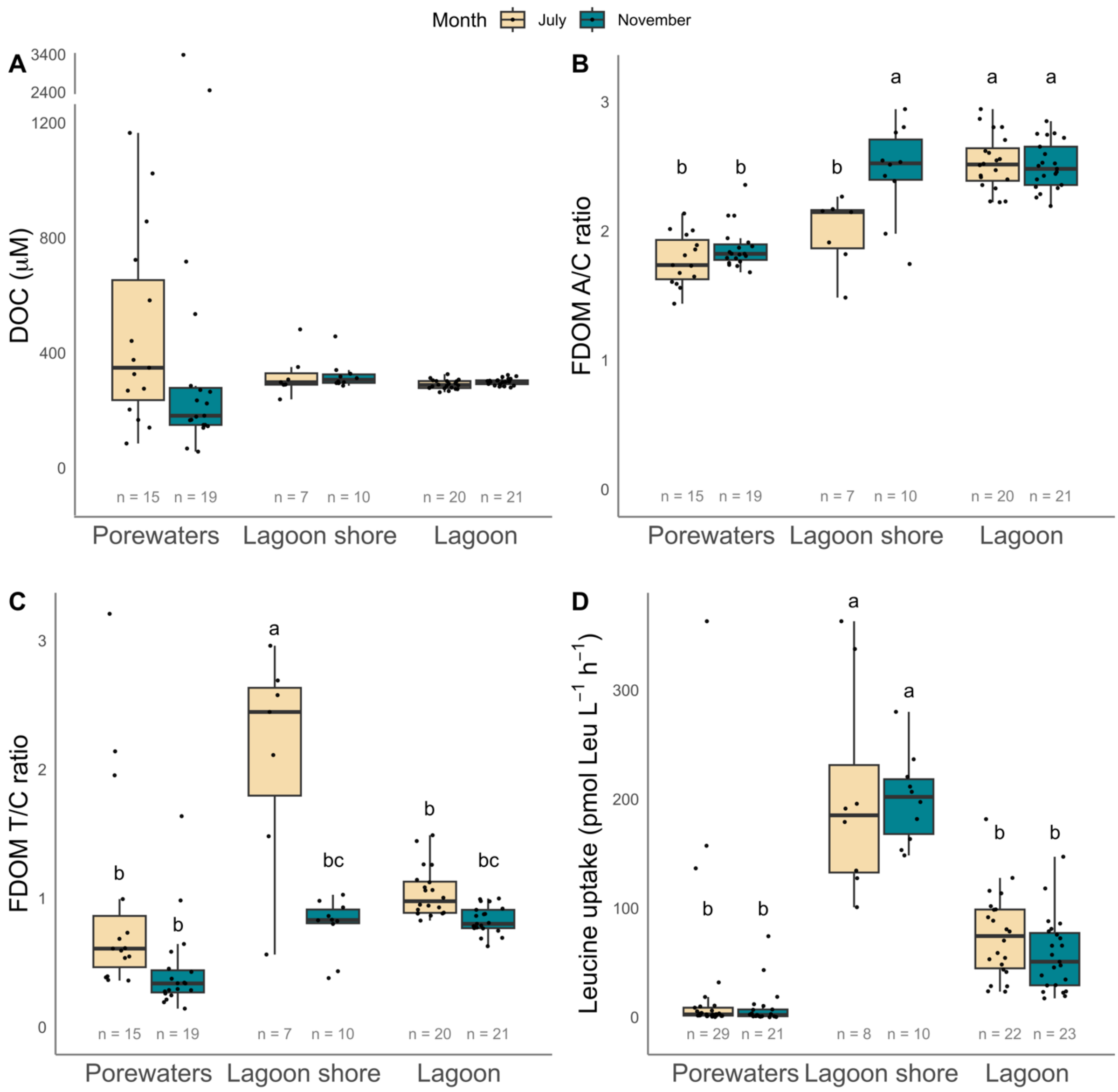

Figure S 3. Main patterns of the geochemical and biological parameters. Boxplots showing the variations of 4 geochemical and biological features according to the water type (porewaters, superficial lagoon shore waters and superficial lagoon waters) and the sampling period (July and November) which is displayed by colors. Significant differences were tested using post-hoc Tukey-Kramer test after unbalanced type III two-way ANOVA and indicated by the letters. Note that in panel A, no significant differences were detected among the tested conditions. FDOM stands for fluorescent dissolved organic matter.

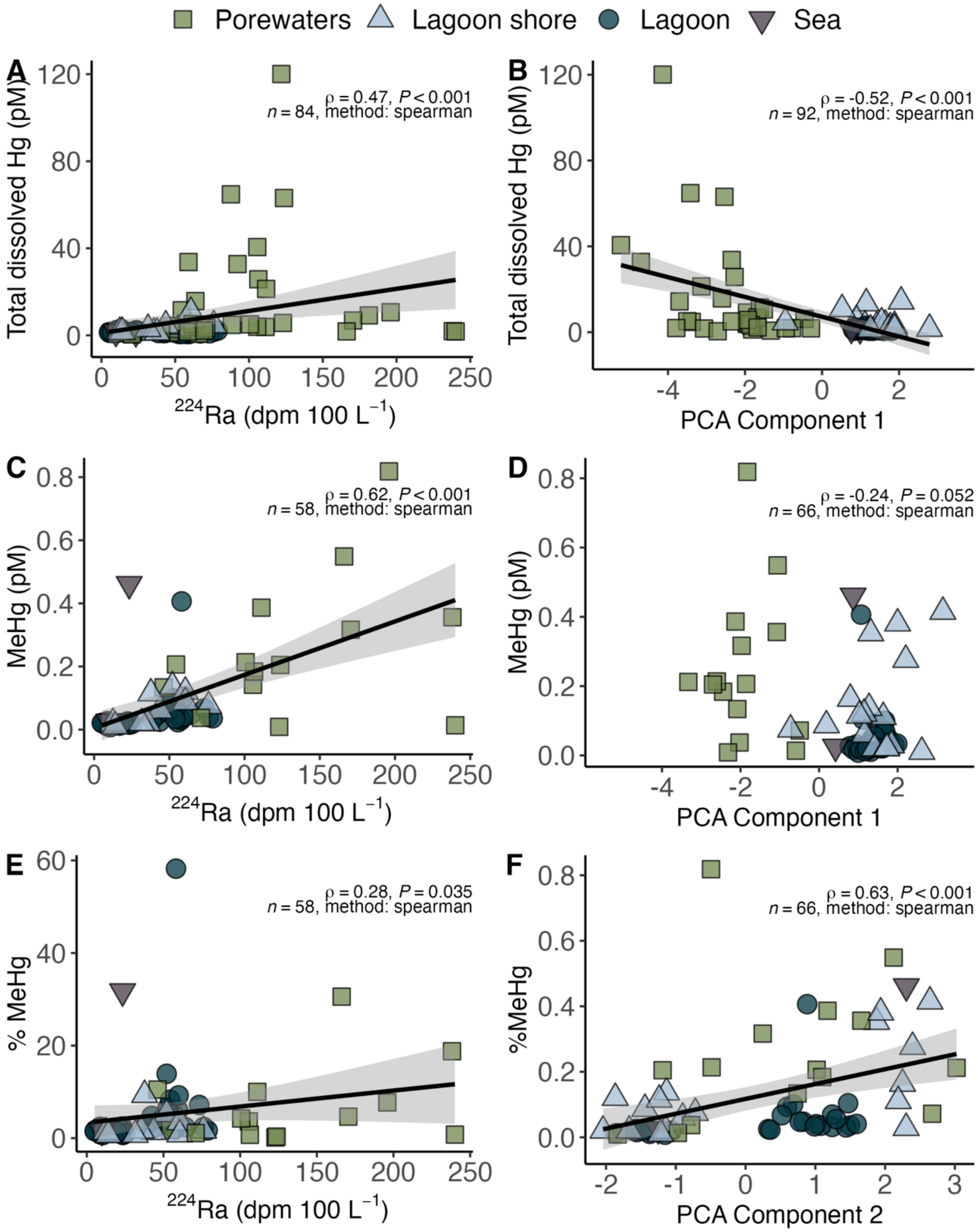

Figure S 4. Non-parametric correlations of Hg species and 224-Ra or PCA components in both sampling periods. A-B) Non-parametric correlations of dissolved total Hg (THg) with the concentration of ^{224}Ra and the first PCA component coordinates and associated statistics. C-D) Non-parametric correlations of dissolved methylmercury (MeHg) with the concentration of ^{224}Ra and the first PCA component coordinates and associated statistics. E-F) Non-parametric correlations of the % of dMeHg compared to dHg with the concentration of ^{224}Ra and the second PCA component coordinates and associated statistics.

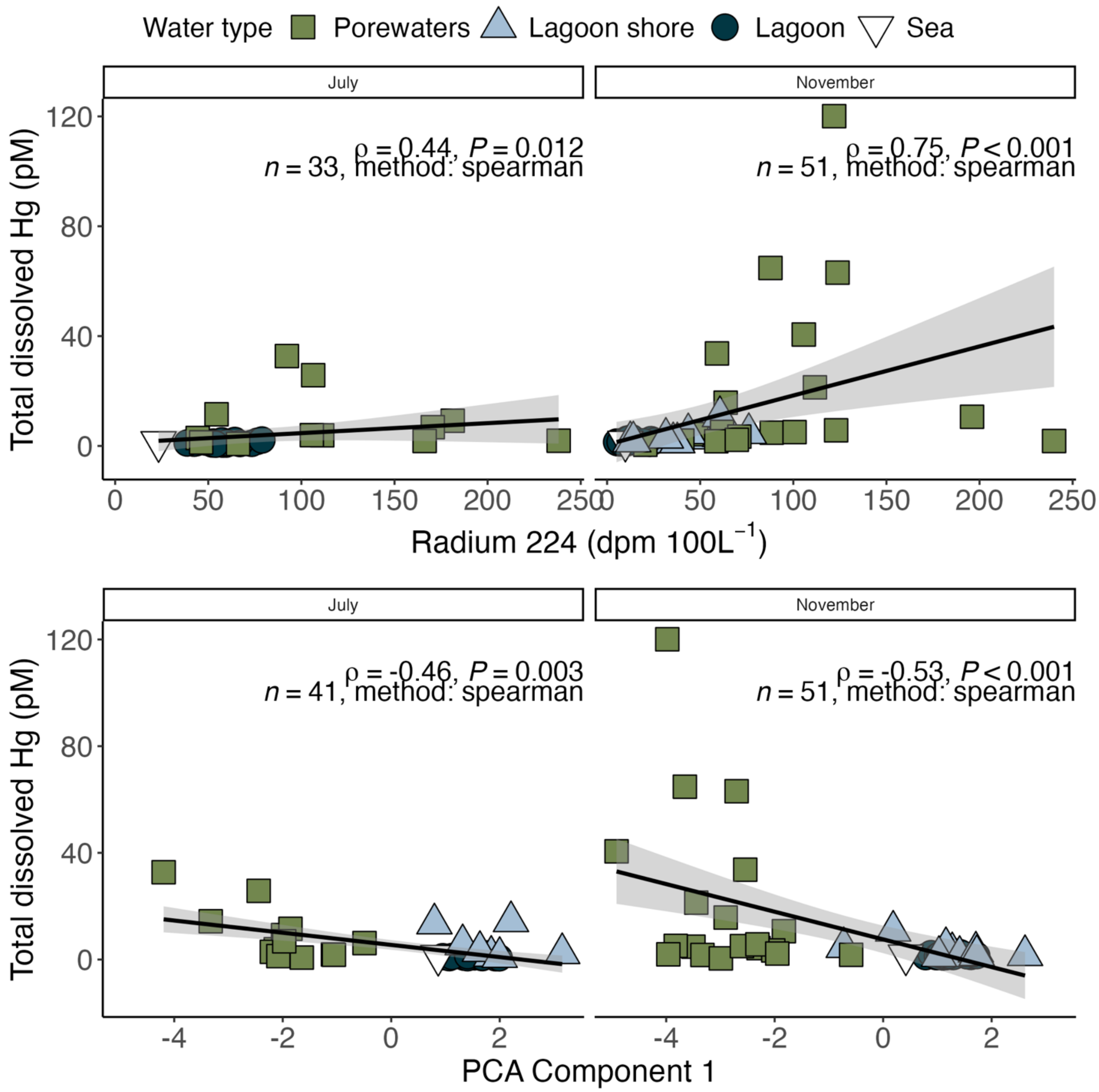

Figure S 5. Linear regressions of total dissolved Hg and SGD proxies (top panel: 224Ra, bottom panel: first component of PCA) separated by sampling periods.

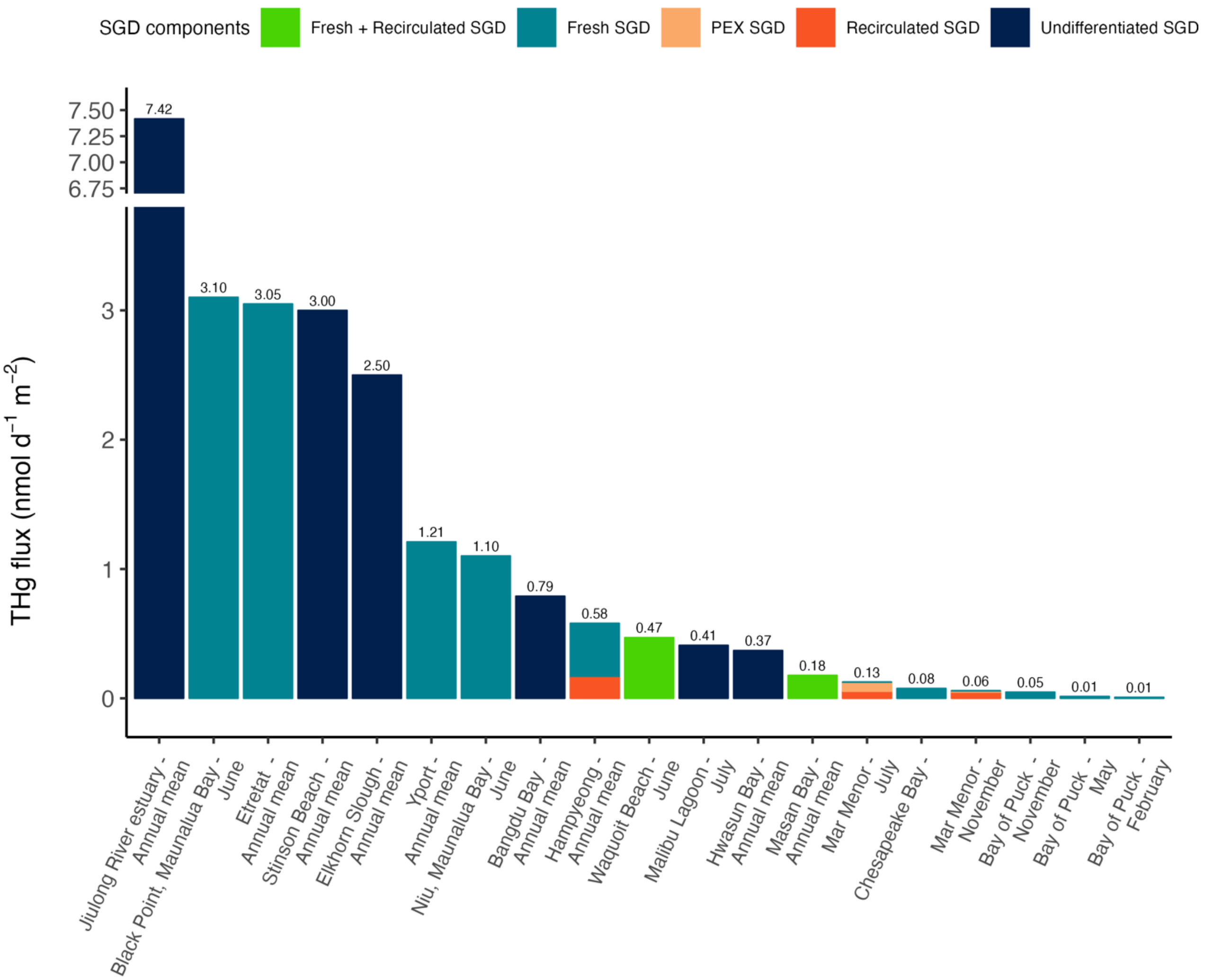

Figure S6. SGDs-based THg fluxes worldwide. The fluxes are reported in nmol day^{-1} m^{-2} and separated by SGD component when available (if not, the category "undifferentiated SGD" was used).

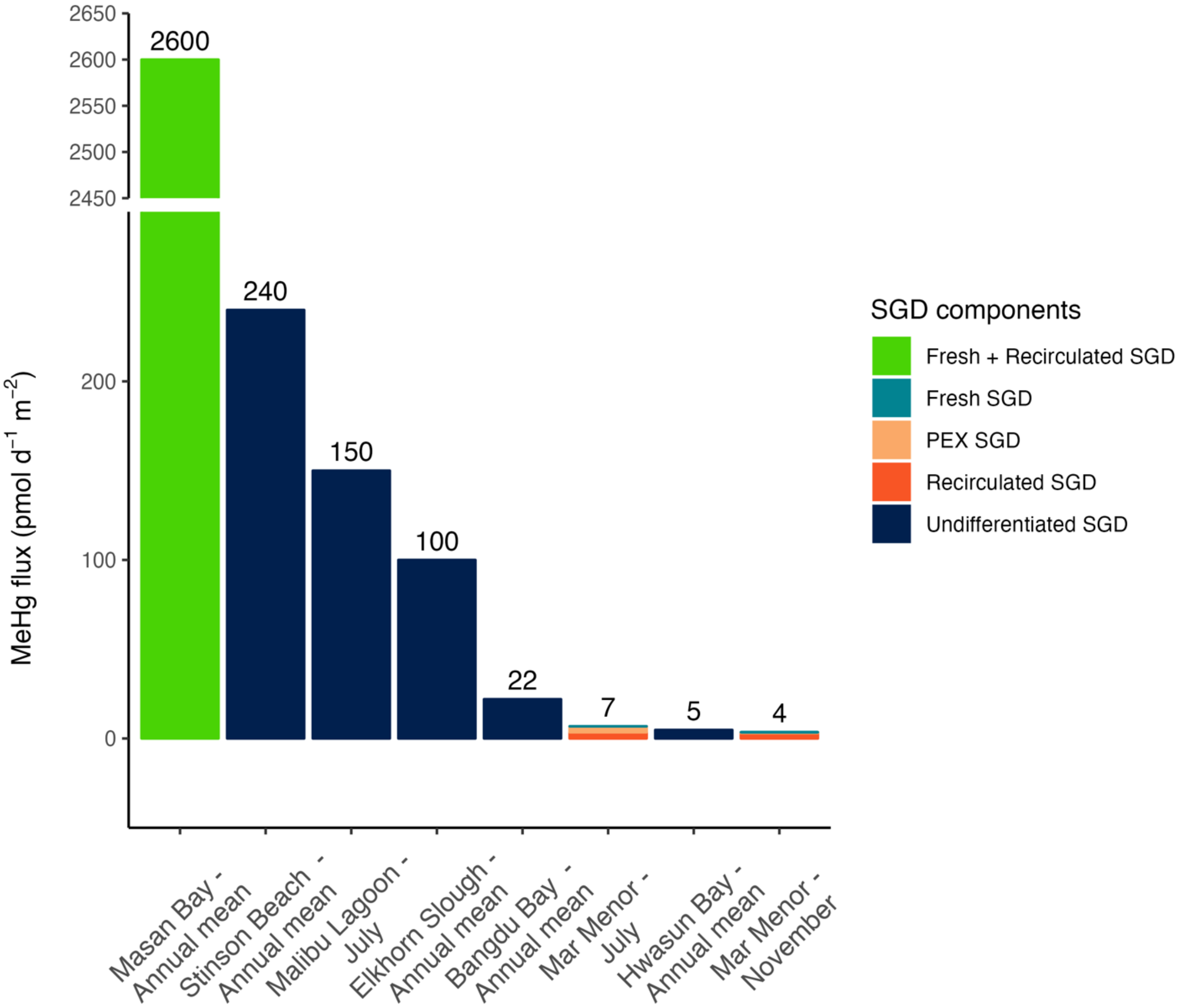

Figure S7. SGDs-based MeHg fluxes worldwide. The fluxes are reported in pmol day^{-1} m^{-2} and separated by SGD components when available.

References

(1) Heimburger, L. E.; Sonke, J. E.; Cossa, D.; Point, D.; Lagane, C.; Laffont, L.; Galfond, B. T.; Nicolaus, M.; Rabe, B.; Van Der Loeff, M. R. Shallow Methylmercury Production in the Marginal Sea Ice Zone of the Central Arctic Ocean. *Sci Rep* **2015**. https://doi.org/10.1038/srep10318.

(2) Torres-Rodriguez, N.; Yuan, J.; Petersen, S.; Dufour, A.; González-Santana, D.; Chavagnac, V.; Planquette, H.; Horvat, M.; Amouroux, D.; Cathalot, C.; Pelleter, E.; Sun, R.; Sonke, J. E.; Luther, G. W.; Heimbürger-Boavida, L. E. Mercury Fluxes from Hydrothermal Venting at Mid-Ocean Ridges Constrained by Measurements. *Nat Geosci* **2024**, *17* (1), 51–57. https://doi.org/10.1038/s41561-023-01341-w.

(3) Alorda-Montiel, I.; Rodellas, V.; Arias-Ortiz, A.; Palanques, A.; Bravo, A. G.; Rodriguez-Puig, J.; Alorda-Kleinglass, A.; Green-Ruiz, C.; Diego-Feliu, M.; Masqué, P.; Gilabert, J.; Garcia-Orellana, J. A Century of Sediment Metal Contamination of Mar Menor, Europe's Largest Saltwater Lagoon. *Mar Pollut Bull* **2025**, *220*, 118347. https://doi.org/10.1016/J.MARPOLBUL.2025.118347.

(4) Romera-Castillo, C.; Lucas, A.; Mallenco-Fornies, R.; Briones-Rizo, M.; Calvo, E.; Pelejero, C. Abiotic Plastic Leaching Contributes to Ocean Acidification. *Science of The Total Environment* **2023**, *854*, 158683. https://doi.org/10.1016/J.SCITOTENV.2022.158683.

(5) Álvarez-Salgado, X. A.; Miller, A. E. J. Simultaneous Determination of Dissolved Organic Carbon and Total Dissolved Nitrogen in Seawater by High Temperature Catalytic Oxidation: Conditions for Precise Shipboard Measurements. *Mar Chem* **1998**, *62* (3–4), 325–333. https://doi.org/10.1016/S0304-4203(98)00037-1.

(6) The MathWorks Inc. MATLAB Version: 9.13.0 (R2022b). The MathWorks Inc.: Natick, Massachusetts, United States 2022.

(7) Garcia-Orellana, J.; Rodellas, V.; Tamborski, J.; Diego-Feliu, M.; van Beek, P.; Weinstein, Y.; Charette, M.; Alorda-Kleinglass, A.; Michael, H. A.; Stieglitz, T.; Scholten, J. Radium Isotopes as Submarine Groundwater Discharge (SGD) Tracers: Review and Recommendations. *Earth-Science Reviews*. Elsevier B.V. September 1, 2021. https://doi.org/10.1016/j.earscirev.2021.103681.

(8) Rodriguez-Puig, J.; Rodellas, V.; Diego-Feliu, M.; Alcolea, A.; Jiménez-Martínez, J.; Alorda-Montiel, I.; Alorda-Kleinglass, A.; Pereira, F.; Manzano, M.; Gilabert, J.; Garcia-Orellana, J. Seasonality of Submarine Groundwater Discharge Pathways in a Coastal Lagoon Revealed by Radium Isotopes: The Importance of Porewater Exchange in Summer. *J Hydrol (Amst)* **2025**, *661*, 133616. https://doi.org/10.1016/J.JHYDROL.2025.133616.

(9) Kirchman, D.; K'nees, E.; Hodson, R. Leucine Incorporation and Its Potential as a Measure of Protein Synthesis by Bacteria in Natural Aquatic Systems. *Appl Environ Microbiol* **1985**, *49* (3), 599–607. https://doi.org/10.1128/AEM.49.3.599-607.1985.

(10) Smith, D. C.; Azam, F. A Simple, Economical Method for Measuring Bacterial Protein Synthesis Rates in Seawater Using 3H-leucine. *Marine Microbial Food Webs* **1992**, *6*, 107.

(11) Rodellas, V.; Garcia-Orellana, J.; Tovar-Sánchez, A.; Basterretxea, G.; López-Garcia, J. M.; Sánchez-Quiles, D.; Garcia-Solsona, E.; Masqué, P. Submarine Groundwater

Discharge as a Source of Nutrients and Trace Metals in a Mediterranean Bay (Palma Beach, Balearic Islands). *Mar Chem* **2014**, *160*, 56–66. https://doi.org/10.1016/J.MARCHEM.2014.01.007.
(12) Cossa, D.; Martin, J. M. Mercury in the Rhône Delta and Adjacent Marine Areas. *Mar Chem* **1991**, *36* (1–4), 291–302. https://doi.org/10.1016/S0304-4203(09)90067-6.
(13) Balls, P. W. Trace Metal and Major Ion Composition of Precipitation at a North Sea Coastal Site. *Atmospheric Environment (1967)* **1989**, *23* (12), 2751–2759. https://doi.org/10.1016/0004-6981(89)90555-6.
(14) Coquery, M.; Cossa, D. Mercury Speciation in Surface Waters of the North Sea. *Netherlands Journal of Sea Research* **1995**, *34* (4), 245–257. https://doi.org/10.1016/0077-7579(95)90035-7.
(15) Coquery, M.; Cossa, D.; Martin, J. M. The Distribution of Dissolved and Particulate Mercury in Three Siberian Estuaries and Adjacent Arctic Coastal Waters. *Water Air Soil Pollut* **1995**, *80* (1–4), 653–664. https://doi.org/10.1007/BF01189718/METRICS.
(16) Stordal, M. C.; Gill, G. A.; Wen, L. S.; Santschi, P. H. Mercury Phase Speciation in the Surface Waters of Three Texas Estuaries: Importance of Colloidal Forms. *Limnol Oceanogr* **1996**, *41* (1), 52–61. https://doi.org/10.4319/LO.1996.41.1.0052.
(17) Coquery, M.; Cossa, D.; Sanjuan, J. Speciation and Sorption of Mercury in Two Macro-Tidal Estuaries. *Mar Chem* **1997**, *58* (1–2), 213–227. https://doi.org/10.1016/S0304-4203(97)00036-4.
(18) Muresan, B.; Cossa, D.; Jézéquel, D.; Prévot, F.; Kerbellec, S. The Biogeochemistry of Mercury at the Sediment–Water Interface in the Thau Lagoon. 1. Partition and Speciation. *Estuar Coast Shelf Sci* **2007**, *72* (3), 472–484. https://doi.org/10.1016/J.ECSS.2006.11.015.
(19) Bloom, N. S.; Moretto, L. M.; Scopece, P.; Ugo, P. Seasonal Cycling of Mercury and Monomethyl Mercury in the Venice Lagoon (Italy). *Mar Chem* **2004**, *91* (1–4), 85–99. https://doi.org/10.1016/J.MARCHEM.2004.06.002.
(20) Allison, J.; Allison, T. *Partition Coefficients for Metal in Surface Water, Soil and Waste*; Washington DC, 2005.
(21) Covelli, S.; Emili, A.; Acquavita, A.; Koron, N.; Faganeli, J. Benthic Biogeochemical Cycling of Mercury in Two Contaminated Northern Adriatic Coastal Lagoons. *Cont Shelf Res* **2011**, *31* (16), 1777–1789. https://doi.org/10.1016/J.CSR.2011.08.005.
(22) Emili, A.; Acquavita, A.; Koron, N.; Covelli, S.; Faganeli, J.; Horvat, M.; Žižek, S.; Fajon, V. Benthic Flux Measurements of Hg Species in a Northern Adriatic Lagoon Environment (Marano and Grado Lagoon, Italy). *Estuar Coast Shelf Sci* **2012**, *113*, 71–84. https://doi.org/10.1016/J.ECSS.2012.05.018.
(23) Bratkič, A.; Ogrinc, N.; Kotnik, J.; Faganeli, J.; Žagar, D.; Yano, S.; Tada, A.; Horvat, M. Mercury Speciation Driven by Seasonal Changes in a Contaminated Estuarine Environment. *Environ Res* **2013**, *125*, 171–178. https://doi.org/10.1016/J.ENVRES.2013.01.004.
(24) Oliveri, E.; Salvagio Manta, D.; Bonsignore, M.; Cappello, S.; Tranchida, G.; Bagnato, E.; Sabatino, N.; Santisi, S.; Sprovieri, M. Mobility of Mercury in Contaminated Marine Sediments: Biogeochemical Pathways. *Mar Chem* **2016**, *186*, 1–10. https://doi.org/10.1016/J.MARCHEM.2016.07.002.

(25) Emili, A.; Acquavita, A.; Covelli, S.; Spada, L.; Di Leo, A.; Giandomenico, S.; Cardellicchio, N. Mobility of Heavy Metals from Polluted Sediments of a Semi-Enclosed Basin: In Situ Benthic Chamber Experiments in Taranto's Mar Piccolo (Ionian Sea, Southern Italy). *Environmental Science and Pollution Research* **2016**, *23* (13), 12582–12595. https://doi.org/10.1007/S11356-015-5281-1,.
(26) Cesario, R.; Hintelmann, H.; O'Driscoll, N. J.; Monteiro, C. E.; Caetano, M.; Nogueira, M.; Mota, A. M.; Canario, J. Biogeochemical Cycle of Mercury and Methylmercury in Two Highly Contaminated Areas of Tagus Estuary (Portugal). *Water Air Soil Pollut* **2017**, *228* (7). https://doi.org/10.1007/s11270-017-3442-1.
